# Phylogeography of the northernmost distributed *Anisocentropus* caddisflies and their comparative genetic structures based on habitat preferences

**DOI:** 10.1002/ece3.7419

**Published:** 2021-03-30

**Authors:** Masaki Takenaka, Saki Shibata, Tomiko Ito, Noriyoshi Shimura, Koji Tojo

**Affiliations:** ^1^ Division of Mountain and Environmental Science, Interdisciplinary Graduate School of Science and Technology Shinshu University Matsumoto Japan; ^2^ Division of Evolutionary Developmental Biology National Institute for Basic Biology Okazaki Japan; ^3^ Department of Biology, Faculty of Science Shinshu University Matsumoto Japan; ^4^ Hokkaido Aquatic Biology Eniwa Japan; ^5^ Nagatsuta Yokohama Japan; ^6^ Institute of Mountain Science Shinshu University Matsumoto Japan

**Keywords:** aquatic insects, comparative phylogeography, habitat preference, lentic and lotic, phylogeny, Trichoptera

## Abstract

Knowledge of the factors that determine the distribution ranges of organisms is necessary to understand their evolutionary and ecological significance and contribution to biodiversity. A very effective mean of studying such factors is to compare the distribution characteristics and genetic structures of closely related species with differing habitat preferences. Freshwater aquatic insects are relatively easy to observe and the basis of their corresponding niche differentiation easier to identify. Freshwater habitats are categorized lotic or lentic water according to flow regime. In Japanese Islands, the genus *Anisocentropus* of the calamoceratid caddisfly, the target group in this study, was morphologically reconfirmed that three species, that is, *Anisocentropus kawamurai*, *A. pallidus,* and *A. magnificus*. Among these, *A. kawamurai* prefers lotic environments and *A. pallidus* is adapted to lentic water habitats. The distribution range of these sister species overlaps within the Japanese Islands. We estimated the phylogeny and the evolutionary history of *Anisocentropus* caddisflies worldwide. We estimated divergence periods by two methods, a single locus with various specimens and multiple loci with reduced numbers of the specimens. As a result, we elucidated the phylogenetic position of Japanese species within the cosmopolitan genus *Anisocentropus*, and also revealed their dual origin. In addition, we demonstrated that the contrasting genetic structures between the sister species distributed in widely overlapping areas were due to differentiation in their respective adapted environmental preferences. Although, in general, it is known that species adapted to lentic water have greater dispersal potential and so are associated with wider distribution areas by means of examining their comparative genetic structures, we revealed a new pattern of genetic locality existing in the genetic structures of the species adapted to lentic water. We then present evidence that suggests the ecological preferences of a species are an important factor in understanding the evolutionary history of that species.

## Graphical abstract

Freshwater aquatic insects are very effective in terms of compare the distribution characteristics and genetic structures of closely related species with differing habitat preferences. We elucidated the phylogenetic position of Japanese species within the cosmopolitan genus *Anisocentropus* and also revealed their dual origin. We then present evidence that suggests the ecological preferences of a species are an important factor in understanding the evolutionary history of that species.

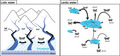

## 1 INTRODUCTION

The distribution range of a species is determined by a combination of factors and is influenced by the particular characteristics of each habitat environment and/or connectivity among habitats. Knowledge of the factors that determine the distribution ranges of organisms is necessary in order to understand evolutionary and ecological significance and contribution to biodiversity (Ellis et al., 2006; Kellermann et al., 2009; Saito & Tojo, 2016a; Tojo et al., 2017). A very effective study of such factors is to compare the distribution characteristics and genetic structures of closely related species with differing habitat preferences (Arbogast & Kenagy, 2001; Emms et al., 2020; Hjalmarsson et al.,  2015; Leaché et al., 2020; Suzuki et al., 2014).

Insects, which are the most diverse group of organisms on earth, evolved while adapting to a variety of niches and environmental conditions (Engel & Grimaldi  2004; Engel et al.,  2013; Misof et al., 2014; Tojo et al., 2017). Among them, freshwater aquatic insects have often attracted attention in studies of niche differentiation on a geographically fine‐scale (Leys et al., 2016; Saito & Tojo, 2016a, 2016b). The differentiation of their habitats based on environmental factors is relatively easy to observe and the basis of the corresponding niche differentiation easier to discuss.

Freshwater habitats can be broadly categorized according to flow regime as either lotic (running water) or lentic (standing water). The majority of aquatic insects inhabit one of these environments, only rarely inhabiting both (Larson, 1997; Ribera & Vogler, 2000). The factor that makes a large difference between lotic water and lentic water species is the degree of connectivity between populations and their corresponding stability. Due to the large differences in characteristics between these two habitat types, the characteristics of the species adapted to each habitat also greatly differ (Abellán et al., 2009; Ribera et al., 2003; Ribera & Vogler, 2000).

**FIGURE 1:**
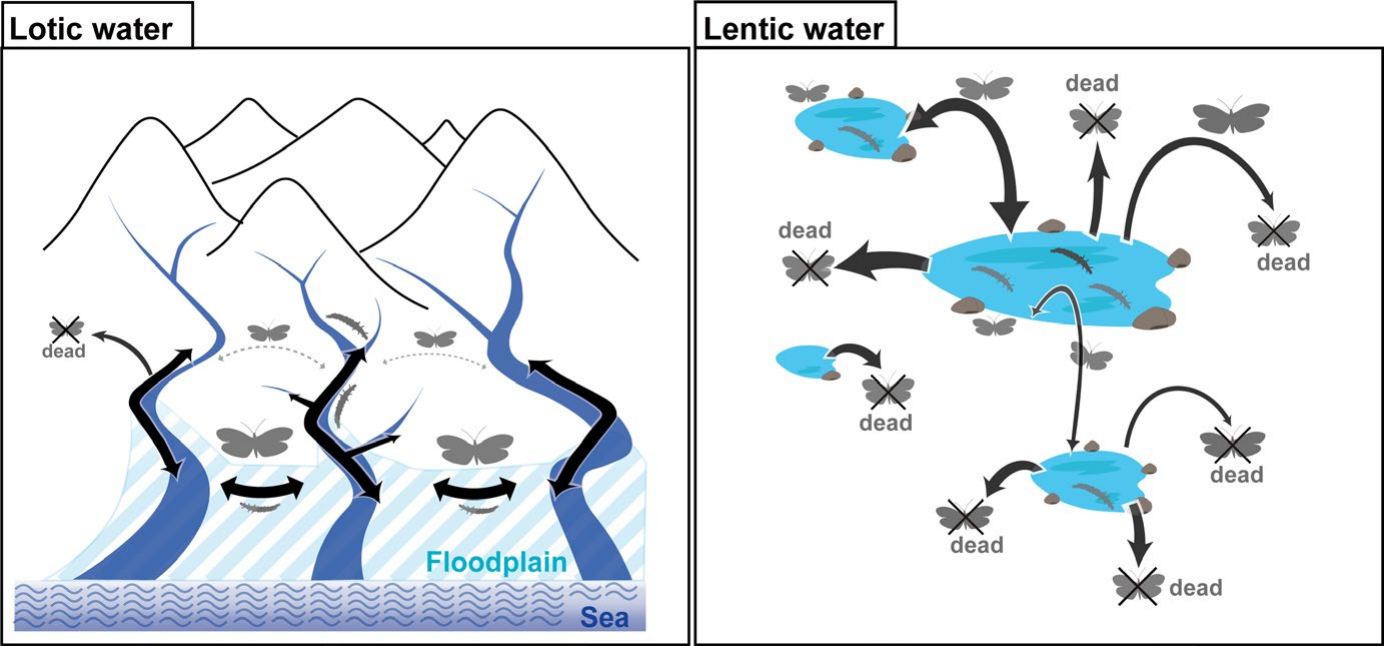
Comparison of dispersion schemes of aquatic insects adapted to lotic and lentic water environments

Lentic water habitats tend to be scattered and isolated having patchy distribution, while lotic water habitats tend to be continuous with the various water habitats connected along the length of a river system. As such, lotic habitats are usually more stable than lentic water habitats (Arribas et al., 2012; Hof et al., 2012). As a result, habitat connectivity and stability are expected to have strong influences on the resultant level of gene flow, genetic drift, and other interpopulation‐level processes (Figure 1; Hughes et al., 2013). Genetic differentiation between populations is likely to be promoted when there are strong restrictions on migration and dispersal between those populations (Hughes et al., 2013; Kato et al., 2013; Takenaka & Tojo, 2019; Takenaka, Tokiwa et al., 2019). Therefore, different scales of gene flow between closely related species should also result in significant differences in the genetic structures of the populations (Abellán et al., 2009; Hughes et al., 2013; Yano et al., 2019).

As a general principle regarding the population structure of aquatic insects, it is considered that species inhabiting lentic environments are more adapted to a relatively higher dispersal capability than those of lotic ones. This is because lentic water environments are isolated and scattered, and as a result, only species with a higher dispersion propensity are likely to have successfully adapted (Bowler & Benton, 2005; Hanski & Gyllenberg, 1997; Hof et al., 2012). Thus, in aquatic insects adapted to lentic water environments, as a result of their typical widespread gene flow, the same genotypes are widely shared; therefore, geographical genetic structure is often not as readily detected (Abellán et al., 2009; Damm et al., 2010; Drotz et al., 2012; Yano et al., 2019).

**FIGURE 2:**
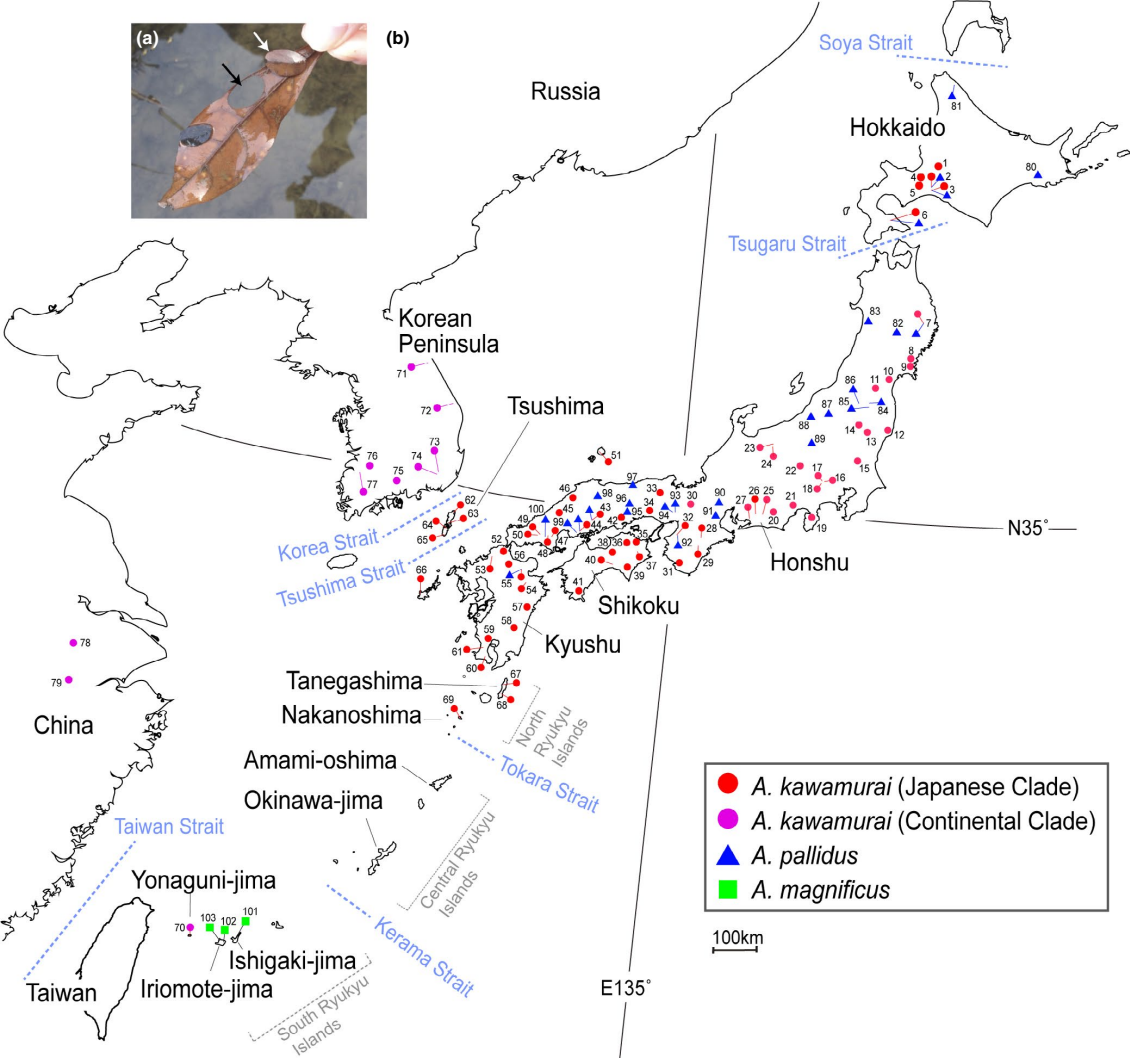
(a) Two larvae of the *Anisocentropus* caddisfly. White arrow indicates a larva with its case, and the black arrow points to the hole where the larva cut its nest material from the leaf. (b) Sampling localities of each species, *Anisocentropus kawamurai*, *Anisocentropus pallidus*, and *Anisocentropus magnificus*. Please refer to Table S1 for specific locality numbers, sample numbers and GenBank accession numbers. The colors correspond to the estimated phylogenetic tree shown in Figure 3

The genus *Anisocentropus* McLachlan, 1863, of the calamoceratid caddisfly is constituted over 90 species and is distributed across the Oriental, Australasian, Afrotropical, Neotropical, Nearctic, and East Palearctic regions (Morse, 2020). The larval cases of the *Anisocentropus* caddisflies are made of two oval and/or rectangular pieces of leaf. The larvae live in such sleeping‐bag like cases and feed on fallen leaves. Thus, their typical habitats are litter packs formed from accumulations of submerged fallen leaves (Ito, 2016; Ito et al., 2012). Ito et al. (2012) reviewed the Japanese species of *Anisocentropus*, and it was morphologically reconfirmed that three species, as follows: (1) *Anisocentropus kawamurai* (Iwata 1927), distributed widely across East Asia, that is, the Japanese Islands, the Korean Peninsula, China, Taiwan, Vietnam, Thailand, and Myanmar (Figure 2; Ito et al., 2012; Takenaka, Shibata et al., 2019). This species prefers lotic environments (*i.e*., running water environments), but also sometimes inhabits lakes, ponds, reservoirs, and stagnant stream water environments. (2) The distribution of *Anisocentropus pallidus* (Martynov 1935) has been recorded within the Japanese Islands (only in Hokkaido, Honshu, and Kyushu, none recorded within Shikoku) and the Russian Far East (Ito et al., 2012; Takenaka, Shibata et al., 2019). The habitats of *A. pallidus* include natural lentic environments, that is, natural lakes, ponds, and swamps (never river streams or artificial ponds: Ito et al., 2012; Takenaka, Shibata et al., 2019). In particular, *A. pallidus* is adapted to lentic water habitats and is only found in isolated stagnant water areas with abundant greenery such as forest edges, swamps, and forest wetlands, and so is never found in large lakes or the side pools of streams banks. (3) The distribution of *Anisocentropus magnificus* (Ulmer 1907) has been recorded on Ishigaki‐jima and Iriomote‐jima Islands in the Southern Ryukyu Islands, and also in the Philippines (Ito et al., 2012). On Iriomote‐jima Island, we collected *A. magnificus* from both lotic and lentic environments of streams and a swamp.

The main objective of this study was to evaluate two hypotheses by comparing the genetic structures of two Japanese species (*A. kawamurai* and *A. pallidus*) with regard to their contrasting habitat preferences. Generally, species adapted to lentic water has higher dispersal capability. However, habitat connectivity and stability are expected to have strong influences on the level of gene flow. There are two major conflicting hypotheses. In this study, we focused on two typical target species with different habitat preferences, of which one, *A. pallidus,* is adapted to strictly lentic environments. This has resulted in a breakthrough in the understanding of comparative phylogeography. We also examined their phylogenetic and evolutionary histories using genetic information on *Anisocentropus* caddisflies obtained from all the continents and islands they inhabit. Our results contribute to accumulating knowledge in one area regarding the temporal diversity of species composition as shaped by shared environments, geological history, idiosyncratic responses to recent climate change, and also a deeper understanding regarding the formation mechanisms of biota within the Japanese islands.

## 2 MATERIALS AND METHODS

### 2.1 Sampling and DNA data

*Anisocentropus kawamurai*, *Anisocentropus pallidus,* and *Anisocentropus magnificus*, the main target species in this study, were collected from almost all recorded areas in the Japanese Islands (Table S1, Figure 2). In addition, for *A. kawamurai*, specimens were also collected from the Korean Peninsula, China, and Thailand. However, the specimens collected from Thailand were old, and so we were not able to obtain good quality gene sequences. In total, 256 specimens of the three *Anisocentropus* caddisflies from 103 localities were collected and analyzed genetically (Table 1, S1, Figure 2). Detailed information on all specimens is shown in Table S1. Most specimens were fixed in 99.5% ethanol in the field, although several were fixed in 70% ethanol and transferred to 99.5% ethanol for long‐term storage.

**TABLE 1:** Basic information on genetic analysis, neutrality tests, and the mismatch distribution analysis of each species of the three targeted *Anisocentropus* caddisflies, based on the mtDNA COI region

Species/Clade	*N*	*H*	*h*	*π*	*GD*	*D*	*F*s
*Anisocentropus kawamurai*	182	70	0.901	0.013	0.014	−1.113	−24.202
Japanese Clade	155	54	0.863	0.004	0.005	−1.855**	−25.902
Continental Clade	27	15	0.943	0.018	0.018	0.342	0.393
*Anisocentropus pallidus*	66	22	0.896	0.007	0.007	−0.552	−4.758
*Anisocentropus magnificus*	8	3	0.464	0.001	0.001	−0.448	−0.478

Abbreviations: *D*, Tajima's *D*; *F*s: Fu's *F*s; *GD*, *p*‐distance within species average; *h*, haplotype diversity; *H*, number of observed haplotypes; *N*, number of examined specimens; *π*, nucleotide diversity.

** *p* < .01.

We added as much sequence data as possible on the mtDNA COI region of *Anisocentropus* caddisflies available from GenBank to analyze the higher phylogenetic relationships among *Anisocentropus* caddisflies around the world. With respect to the out‐groups for our phylogenetic analyses, we used the DNA sequence data of *Molanna* sp. (GenBank accession number: **LC619232, LC619233**) and *Ganonema* spp. (GenBank accession numbers: KX103854, KX104037, KX104287, KX141978). In addition, for our phylogenetic analyses and estimation of divergence periods within *Anisocentropus* caddisflies, we added the DNA sequence data of multiple families as out‐groups; that is, Calamoceratidae and Molannidae which are considered to be sister families to, and Leptoceridae, which is considered to be a sister family to “Calamoceratidae + Molannidae,” and Odontoceridae which is considered to be a sister family to “Calamoceratidae + Molannidae +Leptoceridae” (Thomas et al., 2020, GenBank accession numbers shown in Table S3).

### 2.2 Molecular data collection

The extraction of total genomic DNA and purification of PCR product sequencing were conducted according to the same methods as used in previous studies (Takenaka & Tojo, 2019). Each total genomic DNA sample was used to amplify DNA fragments [the mitochondrial DNA (mtDNA) cytochrome c oxidase subunit I (COI) and the nuclear DNA (nDNA) histone H3 regions] by polymerase chain reaction (PCR) with sets of primers (Table S2). The PCR protocol was shown in Table S2. All sequence data were submitted to the DNA databank of Japan (DDBJ database; Table S1). Sequence alignment and editing were performed for each gene separately using “autostrategy” of MAFFT v7.222 (Katoh & Standley, 2013) and CLC Workbench software (CLC bio, Aarhus, Denmark). The alignments were determined for unique haplotypes and genotypes using the software DnaSP v4.0 (Rozas et al., 2003) prior to subsequent analysis.

Haplotype networks were constructed by the program TCS ver. 1.2.1 (Clement et al., 2000) based on the mtDNA COI region (740 bp) using PopART (Leigh & Bryant, 2015) to visualize and compare genetic structure across genetic barriers for *A. kawamurai* and *A. pallidus*. The extent of genetic diversity (*h*: Nei, 1987) and nucleotide diversity (*π*: Nei & Li, 1979) were investigated using software DnaSP v4.0. We calculated genetic distance (*p*‐distance) between clades using MEGA ver. 6.06 (Tamura et al., 2013) and the *F*st between clades using Arlequin version 3.5 (Excoffier & Lischer, 2010). Population size changes and deviations from neutrality were tested to compare the observed to the estimated mismatch distributions, and measurements of Tajima's *D* statistic (Tajima, 1989) and Fu's *F*s statistic (Fu, 1997) were obtained using Arlequin version 3.5 (Excoffier & Lischer, 2010).

### 2.3 Phylogenetic analyses

Phylogenetic analyses were performed by Bayesian analysis using BEAST 2 ver. 2.4.8 (Bouckaert et al., 2014). The program Kakusan4 (Tanabe, 2007) was used to select appropriate models based on Schwarz's Bayesian information criterion (BIC; Schwarz, 1978). Substitution models were chosen as follows: HKY + G for mtDNA COI and HKY + G for nDNA histone H3. Bayesian MCMC simulations for phylogenetic analyses of Japanese *Anisocentropus* caddisflies were run for 100 million generations, sampling every 10,000 generations for the mtDNA COI region (740 bp), and for 50 million generations, sampling every 1,000 generations for the nDNA histone H3 region. We selected the uncorrelated lognormal relaxed clock and a Yule tree prior. The output files were checked for convergence after removing a 10% burn‐in by examining effective sampling size (ESS > 200) using Tracer v1.6 (Rambaut et al., 2014) and then summarized in Tree Annotator (in BEAST package) before visualizing the resulting tree in FigTree v1.3.1 (Rambaut, 2009).

### 2.4 Estimating divergence periods

To estimate the divergence period for each phylogenetic tree node of *Anisocentropus* caddisflies, we used two methods with a relaxed Bayesian molecular clock analysis was performed with BEAST 2 ver. 2.4.8 (Bouckaert et al., 2014). Although no molecular clock calibrations were attempted due to the lack of appropriate fossil records to strictly conduct divergence periods analyses, we conducted estimated divergence periods using a calibration point estimated as the period when the most recent common ancestor of Molannidae and Calamoceratidae evolved between 78 and 132 Ma (the prior distribution was treated as being lognormal), which was estimated by phylogenetic analyses for Trichoptera based on estimated divergence periods using calibration of an interpretation of the trichopteran fossil record (Thomas et al., 2020). Bayesian MCMC simulations were run for 100 million generations, sampling every 10,000 generations for the mtDNA COI region (589 bp). In addition, to validate the results of estimated divergence periods, we also estimated divergence periods using multiple combined sequence data [the mtDNA COI region, and the nDNA elongation factor 1‐α, carbamoyl‐phosphate synthetase, RNA polymerase II, and isocitrate dehydrogenase regions], but the sample size was small (28 species, 15 genera, 4 families; Table S3). All other settings used were as in the prior, and substitution models are detailed in the above “phylogenetic analysis section.”

### 2.5 Ancestral distribution analysis

To examine the colonization routes of *Anisocentropus* or evolutionary history of Japanese *Anisocentropus* caddisflies, biogeographic history was inferred by the analysis of ancestral area reconstruction using Bayesian binary MCMC (BBM) implemented using RASP 4.0 while applying default parameter settings (Reconstruct Ancestral State in Phylogenies; Yu et al., 2020) and the corresponding likelihood was estimated using model‐based DEC (dispersal, extinction and cladogenesis). We selected biological areas according to “Wallace's Zoogeographic Regions of the World” (Holt et al., 2013); however, the “Sino‐Japanese” [refer to China and Japanese Islands in Holt et al. (2013)] and Palearctic region (only in China and Russia) regions were merged.

## 3 RESULTS

**FIGURE 3:**
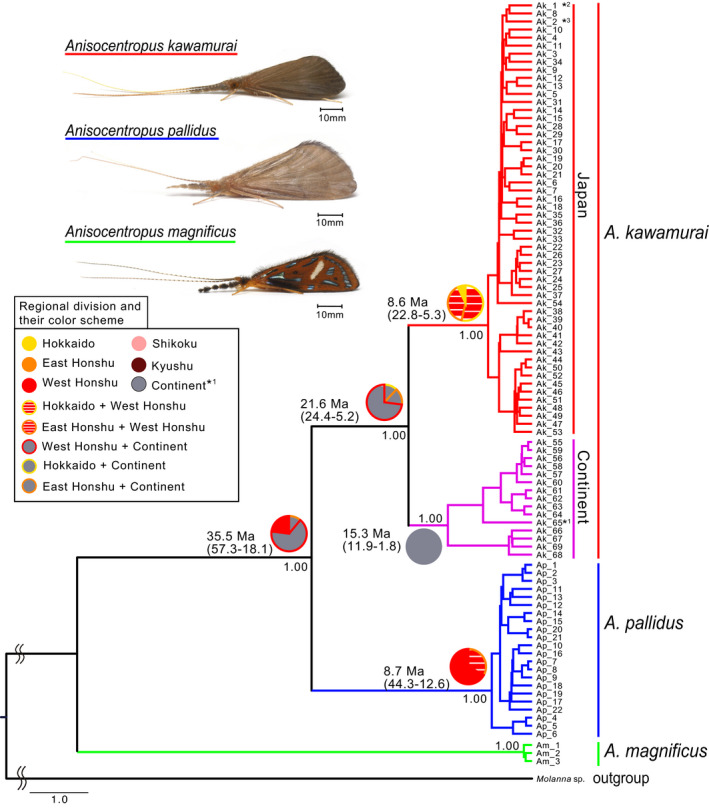
The estimated phylogenetic relationships (Bayes tree) of the Japanese *Anisocentropus* caddisflies based on the sequenced data of the mtDNA COI region (740 bp). The numbers at major nodes indicate posterior probabilities. The pie chart indicates the estimated ancestral distribution areas at each node using the Bayesian binary MCMC methods of RASP. The color scheme of the pie chart for each major node corresponds to the regional division and its color scheme in the box. The Shikoku/Kyushu region was not detected as an ancestral range (*^1^, although Yonaguni‐jima Island is a part of the Ryukyu Islands of Japan, the population of the Island was phylogenetically located within the Continental Clade). The estimated values of divergence periods at major nodes are according to Figures S1 and S2. *^1^, Haplotype observed from Yonaguni‐jima Island. *^2^, Haplotype observed only from Tsushima Island. *^3^, Haplotype observed from Honshu, Shikoku, Kyushu, and Tsushima Island

**FIGURE 4:**
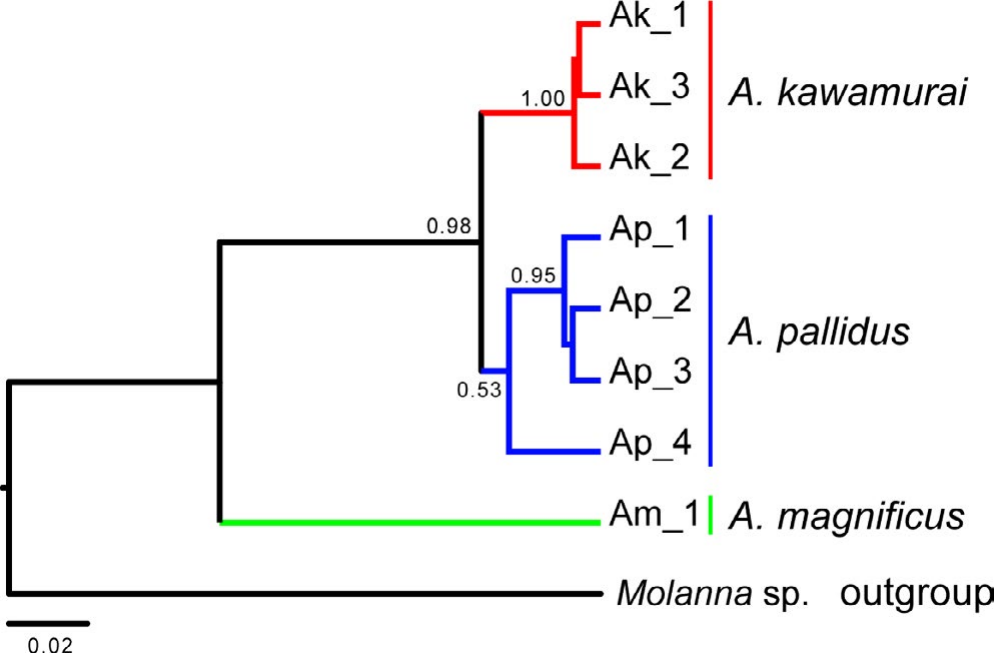
The estimated phylogenetic relationships (Bayes tree) of the *Anisocentropus* caddisflies based on the sequenced data of the nDNA histone H3 region (314 bp). The numbers at major nodes indicate posterior probabilities

The results of our molecular phylogenetic analyses of Japanese *Anisocentropus* caddisflies based on the mtDNA COI region (740 bp) and the nDNA histone H3 region (314 bp) are shown in each Figures 3 and 4. The monophyly of each species, *A. kawamurai*, *A. pallidus,* and *A. magnificus,* was supported. Assessment of potential ancestral distribution ranges using BBM of RASP based on the mtDNA COI region was shown in Figure 3.

**FIGURE 5:**
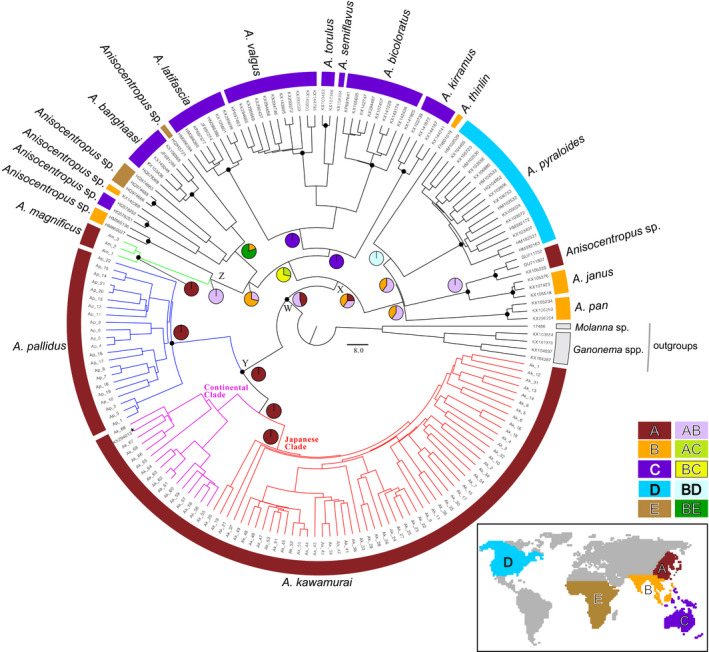
The estimated phylogenetic relationships (Bayes tree) of *Anisocentropus* caddisflies worldwide based on the sequenced data of the mtDNA COI region (589 bp). Black circles at major nodes indicate 1.00 posterior probabilities. The pie charts indicate the estimated potential ancestral ranges using the Bayesian binary MCMC methods of RASP (Yu et al., 2020). Each node of W, X, Y and Z, which is considered to be particularly important, is discussed within the text in detail. *, KX294013 is registered from Russia as *A. pallidus*, but this specimen seemed to be a misidentification of *A. kawamurai*

Subsequently, we assessed the potential ancestral distribution ranges of *Anisocentropus* globally using their phylogenetic tree. As a result of that analysis, it was supported initial distribution was within the East Asian region (Figure 5). In addition, it was clarified that *A. kawamurai* and *A. pallidus* form a monophyletic clade and are genetically largely differentiated from other *Anisocentropus* caddisflies. Species inhabiting the East Asian and South‐East Asian regions were found to be in a mosaic pattern at various sites within the phylogenetic tree, thus demonstrating a high degree of species and genetic diversity across the Asian region. *Anisocentropus magnificus*, which inhabits the Yaeyama Islands of Japan, largely genetically differentiated from the other two Japanese species, and rather was located within a clade composed of South‐East Asian, Australian, and African lineages (Figures 3, 4, 5).

Regarding the intraspecies phylogenetic relationships of *A. kawamurai*, the phylogenetic tree based on the mtDNA COI region showed a large degree of the genetic differentiation between the populations of the Japanese Islands and those of the Eurasian Continent (Table 2, Figure 3). The population of Yonaguni‐jima Island (i.e., site No. 70 in Figure 2), which is a part of the Japanese Islands, was genetically located as being from within the Eurasian Continental clade (Haplotype Ak‐65; Figure 3). A Russian specimen, which was registered as *A. pallidus* in GenBank, was also included within the Eurasian Continental clade of *A. kawamurai* (KX294013, Figure 5; this sample is considered to be a taxonomic misidentification).

**TABLE 2:** Genetic distances (*p*‐distance) between the targeted *Anisocentropus* caddisflies, based on the sequence data of the mtDNA COI region

	*A. kawamurai* JPN Clade	*A. kawamurai* CNT Clade	*A. pallidus*	*A. magnificus*
*Anisocentropus kawamurai*				
Japanese Clade		0.7972	0.9012	0.9591
Continental Clade	0.0383		0.8563	0.8914
*Anisocentropus pallidus*	0.0572	0.0300		0.9455
*Anisocentropus magnificus*	0.1164	0.1280	0.1162	

Note

Upper diagonal, pairwise *Fst* values; lower diagonal, *p*‐distance.

The genetic diversity (i.e., both haplotype diversity and nucleotide diversity) of the populations of the Eurasian Continent of *A. kawamurai* was found to be higher than that of the populations of the Japanese Islands (Table 1, Figure 3). In addition, recent expansions of the effective population sizes of the Japanese lineages were clearly indicated (see Tajima's *D* and Fu's *F*s; Table 1). As for the two species of *A. pallidus* and *A. magnificus*, analyses were only conducted on the Japanese populations (Figures 3 and 4).

### 3.1 Estimating divergence periods of *Anisocentropus* caddisflies

To determine the phylogenetic positioning and divergence periods of the three Japanese *Anisocentropus* species, we conducted phylogenetic analyses for *Anisocentropus* caddisflies on a worldwide scale, using all available GenBank datasets (Figures 5, S1 and S2). In addition, we estimated the divergence period at each node of *Anisocentrupus* caddisflies based on each calibration point at which the divergence period of the most recent common ancestor of the two closely related families (Molannidae and Calamoceratidae) had evolved between 78 and 132 Ma (Thomas et al., 2020). For this result, the origin of *Anisocentropus* was estimated as being 75.3 (116.9–42.6 Ma), that is, Cretaceous to Paleogene (Figure S1). In addition, to validate the results of estimated divergence periods in Figure S1, we also estimated the divergence periods using multiple combined sequence data. For this result, the origin of *Anisocentropus* was estimated as being 74.1 (122.8–35.1 Ma; Figure S2). Analyses using two datasets produced almost the same divergence ages.

### 3.2 Comparing genetic structures of sister species adapted to different environments

**FIGURE 6:**
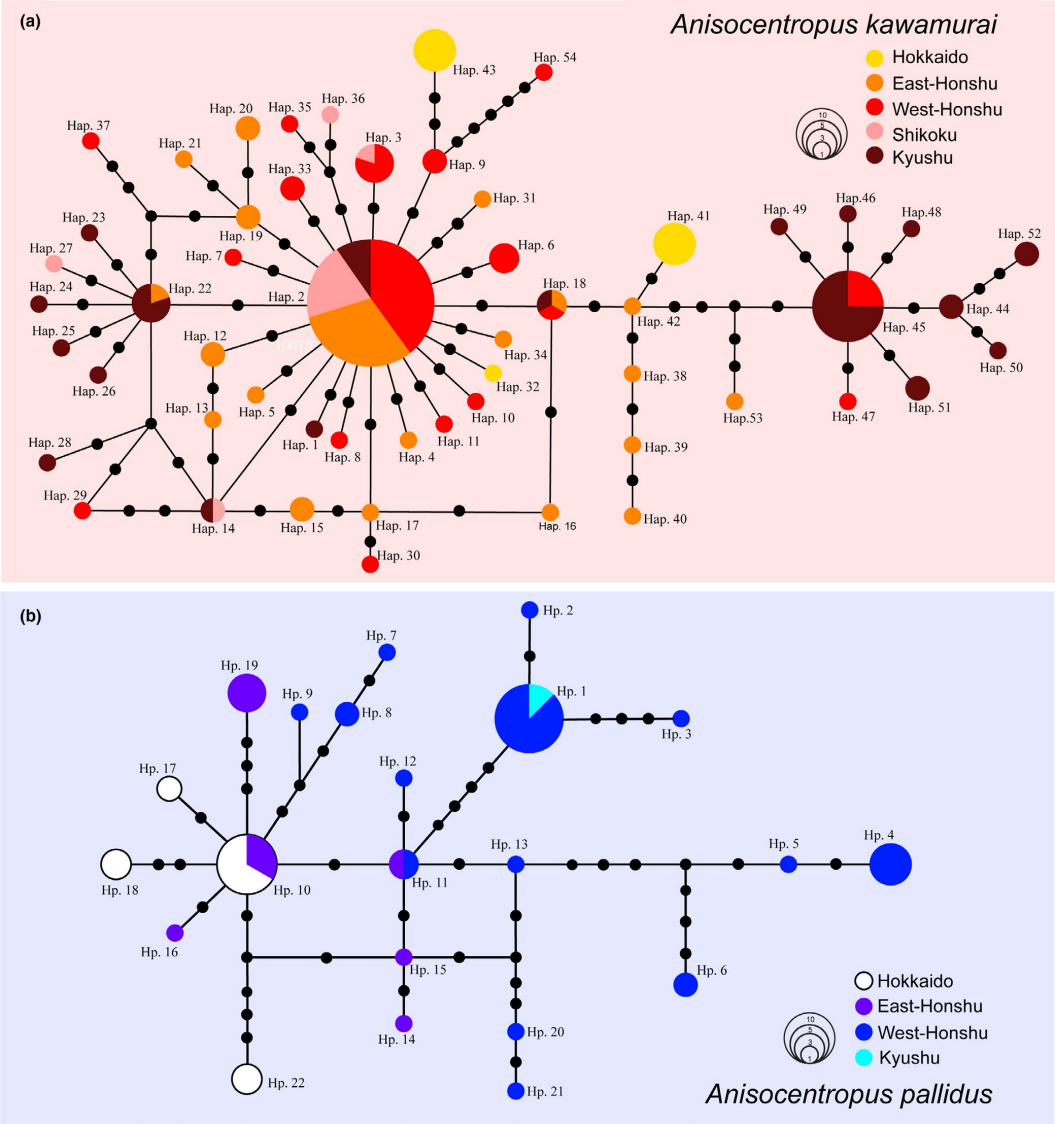
Haplotype networks of the Japanese *Anisocentropus kawamurai* (A) and *Anisocentropus pallidus* (B) based on the sequenced data of the mtDNA COI region (740 bp). These haplotype numbers correspond to the numbers (names) of OTUs of the phylogenetic tree in Figure 3

Here, we compared the genetic structures of two *Anisocentropus* species, *A. kawamurai* and *A. pallidus*, which have differing habitat preferences. Regarding the mtDNA COI region, a total of 70 haplotypes were identified from 183 specimens collected from 79 sites in *A. kawamurai*, and a total of 22 haplotypes were identified from 66 specimens collected from 28 sites in *A. pallidus* (Table 1, Table S1, Figure 6). The haplotype diversity (*h*) and the nucleotide diversity (*π*) values of these species are shown in Table 1. The haplotype network of *A. kawamurai* was constructed resulting in a typical “star”‐like network, indicating that many other haplotypes were derived from these two dominant haplotypes (Figure 6a). The haplotype network of *A. pallidus* was constructed whereby the haplotypes observed were found to be scattered among many other presently unobserved intermediate haplotypes (Figure 6b).

## 4 DISCUSSION

### 4.1 Evolutionary history of *Anisocentropus* caddisflies

The level of species diversity for *Anisocentropus* is known to be higher within the Oceania region (Morse, 2020). The sequence data of the mtDNA COI region of *Anisocentropus* caddisflies in various regions were registered in GenBank following a previous study (Zhou et al., 2016) as part of the Trichopteran DNA barcoding project. Of significance in this study, we added gene sequence data for *Anisocentropus* caddisflies in Asia, especially in Japan. We then estimated the phylogeny and the evolutionary history of *Anisocentropus* caddisflies worldwide, and we aimed to clarify the evolutionary history and phylogenetic positions of the Japanese species.

Although this result may have been influenced by sampling bias due to the number of Japanese specimens being extremely large and the monophyly of the node W was not so strongly supported, it was found that *A. kawamurai* and *A. pallidus* might have diverged at the earliest node from other species in our dataset (i.e., node W in Figure 5).

Then, as a result of our estimation of the ancestral range of *Anisocentropus* caddisflies worldwide, it seems probable that their ancestral range is more correctly estimated to be within the East and/or South‐East Asian regions rather than Australia (i.e., node X in Figure 5), because the level of species diversity is known to be higher than these regions in Australia. This result that the origin of *Anisocentropus* caddisflies is in the Asian region is clearly a subject that should be thoroughly studied in the future. In addition, as the number of species described within South‐East Asia has been increasing in recent years (Suwannarat et al., 2018), it is important to accumulate further knowledge within the Asian regions in order to properly assess the degree of species diversity within this group.

As a result of estimated divergence period based on the mtDNA COI region, the origin of *Anisocentropus* was estimated to be 75.3 (116.9–42.6) Ma (Figure S1). This analysis included many specimens from various regions, although it used only a single locus to estimate divergence dates. As a subsequent step, in order to confirm the accuracy of this age, estimates of the divergence periods of *Anisocentropus* were also analyzed based on sequence data of five loci including regions of mitochondrial and nuclear DNA although the number of samples was smaller (Figure S2). As a result, the estimated divergence period between *Anisocentropus* and *Ganonema,* which are sister genera, was also estimated as having similar a divergence age using both analyses. While it is possible that the estimated divergence period using calibration based upon a specific node may result in overestimation, the calibration point used in this study was estimated based on the divergence period between Calamoceratidae and Molannidae (also sister families) as estimated in a previous study (Thomas et al., 2020 ), which thoroughly examined the phylogeny of Trichoptera based on multiple loci and including almost all Trichopteran families, giving estimated divergence periods using calibration based on many nodes and including a review of fossil records. Therefore, it is considered that the risk of significant overestimation of the divergence periods was minimized, and we consider that the estimated divergence periods were reasonably estimated in this study.

The origin of the diversification of *Anisocentropus* caddisflies was estimated to be around the time at which the division of the “Supercontinent Pangea” was in progress, as the prototype of each current continent began to be formed (Paleomap PaleoAtlas for GPlates: https://www.earthbyte.org/paleomap‐paleoatlas‐for‐gplates/; Earth View: http://media.hhmi.org/biointeractive/earthviewer_web/earthviewer.html).

The distribution range of a species is influenced by the particular characteristics of each habitat's environment and/or the connectivity among habitats (Arbogast & Kenagy, 2001; Emms et al., 2020; Múrria & Hughes, 2008; Okamoto & Tojo, 2021). Therefore, it is possible that taxa having similar distribution areas may have experienced a similar evolutionary history. For example, the distribution areas of the family Stenopsychidae are similar, and *Stenopsyche* has spread into wide areas of the Asian region of the Northern Hemisphere. As such their origin may be considered to be Gondwanaland based on their current distribution area and that the Japanese Islands and their surrounding areas correspond to the northern and furthermost distribution limits of this group (Morse, 2020; Nozaki et al., 2016; Saito et al., 2018).

The distribution areas of Stenopsychid and *Anisocentropus* caddisflies are similar. It is considered that these two caddisfly groups have been strongly influenced by the same geohistorical events since the division of the supercontinent. It was presumed that the caddisflies of *Stenopsyche* had their highest genetic diversity within the Southeastern Asian region and then speciated as they expanded distribution to the north and into the East Asian region (Saito et al., 2018). *Anisocentropus* caddisflies are also considered to have originated in the South‐East Asian region (node X in Figure 5), similar to *Stenopsyche* caddisflies. Although the node Y is shown to have the high posterior probability of the East Asian clade, the node W is likely to have a sample bias toward East Asia. So, it is highly likely that it originated in South‐East Asia.

However, there were differences identified between these two caddisflies in the process of their distributional expansion and phylogeographic evolution within the Asian region. The two species inhabiting the Japanese Islands and their surrounding areas (i.e., *A. kawamurai* and *A. pallidus*), which correspond to the northern limit of the distribution area of *Anisocentropus* caddisflies, were genetically differentiated from all other *Anisocentropus* species worldwide. It is considered that these differences between the genetic structures of *Stenopsyche* and *Anisocentropus* are due to the differences in their habitat preferences. Larvae of *Anisocentropus* caddisflies prefer leaf litter packs formed from accumulations of submerged fallen leaves in ponds or/and pools within streams (Ito, 2016; Ito et al., 2012), while larvae of *Stenopsyche* caddisflies prefer riffle environments and gravel riverbeds in flowing water (Saito et al., 2018). Thus, it is speculated that the differences in habitat preferences have influenced phylogeographic evolutionary history and the genetic structures of these two caddisfly groups (Abellán et al., 2009; Emms et al., 2020; Ribera et al., 2003).

### 4.2 The evolutionary history and distribution ranges of three Japanese *Anisocentropus* caddisflies

As a result of our molecular phylogenetic analyses, each of the three species, *A. kawamurai*, *A. pallidus,* and *A. magnificus*, was evaluated as being clearly genetically differentiated clades, which agreed with a previous morphological taxonomical study (Ito et al., 2012). The speciation between *A. kawamurai* and *A. pallidus* was estimated to have occurred 35.5 (57.3–18.1) Ma, when the Japanese Islands were located to the Eurasian Continent as a landmass on the eastern edge of the continent (Martin, 2011; Otofuji et al., 1985), based on the analysis of the estimated divergence periods (Figure 3). The lineage of many animals that inhabit the present Japanese Archipelago is thought to have diverged from a common ancestor that inhabited the landmass that forms the current Japanese Islands when it was still a part of the continent (Motokawa & Kajihara, 2017; Tojo et al., 2017). Both *A. kawamurai* and *A. pallidus* are also considered to be typical examples of having such an eastern Eurasian Continental origin.

On the other hand, it was also shown in our study that one more Japanese species, *A. magnificus* constitutes a monophyletic clade with another species distributed in Southeast Asia and Oceania regions, and it is largely genetically differentiated from the other two Japanese *Anisocentropus* species. The distribution range of *A. magnificus* includes the Yaeyama region (i.e., Ishigaki‐jima and Iriomote‐jima Islands) and the Philippine Islands. Although we could not collect specimens from the Philippines, it is considered that the population of the Yaeyama region may have derived from those of the Philippine Islands, like several organisms (Tseng et al., 2018).

Therefore, we suggest that Japanese *Anisocentropus* caddisflies have, at least, dual origin, one from East Asia and the other from South Asia. It also became clear that these two lineages have had contact with the Ryukyu Islands. In the future, it will be necessary to carry out research in both the Philippines and Taiwan.

### 4.3 Genetic structures of *A. kawamurai*

#### 4.3.1 Genetic differences between the Japanese Islands and the Eurasian Continent

We included genetic data for *A. kawamurai* in our genetic analyses from a wide area of the Japanese Islands and the Eurasian Continent (i.e., the Korean Peninsula, the Chinese Mainland). Cases of genetic differentiation between the organisms of the Japanese Islands and the Eurasian Continent were reported in many species (Motokawa & Kajihara, 2017; Tojo et al., 2017). Insects are no exception, suggesting such differentiation patterns have a relationship to the geological events which occurred during the division of the Japanese Islands from the Eurasian Continent (cf. Tojo et al., 2017). This study also showed large genetic differentiation of *A. kawamurai* between the Japanese Islands and the Eurasian Continent. In addition, the sequence data of *A. pallidus* from Russia registered in GenBank have been identified as within the continental phylogeographic clade of *A. kawamurai*. To date, only *A. pallidus* are recorded from Russia, so it is possible that it was treated as species *A. pallidus* with insufficient consideration. However, this study also demonstrated an increased likelihood that *A. kawamurai* could potentially inhabit Russia. If this conjecture is correct, it will result in a newly recorded species in Russia, but strictly speaking, the morphological characteristics of a specimen of the population from which the genetic data were derived, and which we used in our genetic analysis needs to be scrutinized.

The divergence period between the Japanese and the Eurasian Continental lineages of *A. kawamurai* was estimated as 21.6 (5.2–24.4) Ma. This agrees with the geologically estimated period of the Japanese Archipelago separating from the Eurasian Continent (Otofuji et al., 1985). It therefore indicates that there has been no subsequent dispersion and gene flow since the separation of the Japanese Archipelago.

Genetic diversity (i.e., haplotype and nucleotide diversity) within the continental populations was higher than that of the Japanese populations. One of the hypotheses proposed that the ancestral Japanese population originated as a part of the continental lineages and thereafter evolved independently within the Japanese Islands. Another hypothesis is that it is generally more difficult to maintain genetic diversity on small islands, as there are smaller population sizes, than on continents. Also, the risk of bottlenecks and extinction is higher than in continental populations (MacArthur & Wilson, 2001), and it is possible that the impacts of glacial–interglacial cycles were stronger than in the continental populations. In our previous study's ecological niche model analysis, stable habitable environments (potential distribution regions) were estimated to be over a wider area on the continent than in the Japanese Islands during the glacial periods (Takenaka, Shibata et al., 2019). Therefore, it is considered that the population decline on the continent during the cold glacial periods would have been lower than that within the Japanese Islands.

Although it was only evaluated for the Japanese populations that the population size expansion was relatively recent using neutrality tests (Tajima's *D* and Fu's *F*s tests), it was estimated that the divergence periods of the Japanese populations compared with the continental populations are older, when the proto‐Japanese Islands separated from the eastern edge of the Eurasian Continent. It is also considered that population declines during the glacial periods were followed by expansions during the following warmer periods. From haplotype network analysis, it appears that there may have been multiple refugia during the glacial periods. No geographical genetic structures were detected within the continental populations. Therefore, the continental lineage has maintained gene flow over a wide area along with a corresponding high degree of genetic diversity.

The Tsushima Islands are a very important area when discussing biota formation within the Japanese Islands (Tojo et al., 2017). Various organisms that migrated to Japan via the land bridge, of which the Tsushima Islands form a part, are well known [e.g., *Carabus procerulus* (Kim et al., 2000), *Ephoron shigae* (Sekiné et al., 2013, 2015)]. In *A. kawamurai*, it was clearly shown that the Tsushima population has a genetic structure closer to that of Japanese populations than Korean (asterisks 2 and 3 in Figure 3). In addition, since the genetic differentiation between the Japanese Islands and the Eurasian Continent of *A. kawamurai* occurred at the edge of the continent in an early period, and the Tsushima population is genetically positioned as a part of the Japanese clade, it is considered that a part of the Japanese population migrated to the Tsushima Islands from the Japanese Islands, rather than having migrated from the Continent to the Japanese Islands.

#### 4.3.2 Japanese southwestern populations of *A. kawamurai*

Only the population of Yonaguni‐jima Island, which is located at the westernmost reaches of Japan, was genetically positioned within the Eurasian Continental clade. This is because Yonaguni‐jima Island was connected to the continent via Taiwan for a long time due to events in geological history (Kato et al., 2013; Koizumi et al., 2014; Osozawa et al., 2017). It can be seen from the Bathymetrical map (marine topographical map) around the Japanese Islands that the South Ryukyu Islands and Taiwan, when the sea level drops, become connected by land bridges. Nondistribution of *A. kawamurai* within the Central Ryukyu Islands is also consistent with the topographical map of the seafloor.

Interestingly, *A. kawamurai* is not distributed on either Ishigaki‐jima or Iriomote‐jima Islands, even though they are within the same Southern Ryukyu Island chain. Such a distribution pattern is also observed in several other caddisfly species there (Ito, 2017). This unique distribution pattern may be related to their interspecific interaction with *A. magnificus* of South Asian origin. The South Ryukyu Islands are an interesting area, within which is observed the borderline between the distributions of *A. kawamurai* and *A. magnificus*.

### 4.4 Comparative genetic structure between sister species with different preference environmental characteristics

The distribution range of *A. kawamurai* (adapted to lotic environments) and *A. pallidus* (adapted to lentic environments) overlaps within the Japanese Islands, which is a rare case in which two species inhabit an area sympatrically (Takenaka, Shibata et al.,  2019). In this study, there were only seven sites in which both species were sympatrically collected. *Anisocentropus pallidus* inhabits only isolated stagnant water areas with a high degree of naturalness and has never been observed inhabiting large lakes or the side pools of streams. Therefore, the habitat of *A. pallidus* itself has a fairly limited distribution.

As a result of the molecular analysis in this study, the speciation of these two species of *A. kawamurai* and *A. pallidus* was estimated to have occurred ca. 35.5 Ma. Therefore, it is considered that the speciation due to niche differentiation occurred at the continental time when the Japanese archipelago was still located on the eastern edge of the Eurasian Continent (Martin, 2011; Otofuji et al., 1985). Within the current Japanese Islands, although their distribution areas overlap widely, their habitat preferences seem to still be maintained.

The genetic structure of an organism is determined by the interaction of a complex mix of factors such as preferred habitat characteristics, historical background, barriers to migration, and geological history (Arbogast & Kenagy, 2001; Emms et al., 2020; Saito & Tojo, 2016b). The two species, *A. kawamurai* and *A. pallidus*, are suitable groups for elucidating the relationship between the difference in habitat preference and the corresponding genetic structure. A different genetic structure was observed between each of the species, *A. kawamurai* and *A. pallidus*.

The genetic structures showed a contrasting trend. In the haplotype network of *A. kawamurai*, two major dominant haplotypes were observed, and a “star” structure was formed around these major haplotypes. On the other hand, from the genetic structure of *A. pallidus*, many intermediate haplotypes were observed; in addition, we also detected the existence of hypothetical haplotypes that were not sampled or extinct, and this network shape indicated that a stable population structure has been maintained for a long time. Also, the genetic structure and neutrality tests indicated that the population structure of *A. pallidus* is a more stable population structure. It is considered that such a difference in their genetic structural patterns is due to the difference in their habitat preferences. Therefore, since the lotic water environment is geographically more continuous, the potential for gene flow between populations of species adapted to lotic water conditions exists over a wider area. In general, species adapted to environments, which are scattered and isolated with a patchy distributing, have a limited potential for gene flow. Therefore, there is a higher potential for genetic differentiation between different populations, as a result of the restricted level of gene flow and also for subsequent genetic drift and other interpopulation‐level differentiate processes (Hughes et al., 2013; Water et al., 2020). Meanwhile, some other intermediate haplotypes that would have likely existed in the past may have become extinct due to the effects of unstable environments, whereas, when such habitats are stable for a long period, it can facilitate the accumulation of genetic differentiation within its population from other populations. Many studies focused on organisms adapted to patchily distributed habitats and/or low dispersion species reported many intermediate haplotypes, high nucleotide diversity values, a high number of mutations, and a high degree of genetic differentiation between populations (e.g., Okamiya et al., 2018; Su et al., 1996; Takenaka & Tojo, 2019).

These contrasting genetic structures are similar to those observed between the genetic structures of species in which their habitats are positioned continuously as opposed to those in which their habitats have a patchy distribution, and/or between species with differing degrees of dispersal ability (Drotz et al., 2012; Hjalmarsson et al., 2015; Ikeda et al., 2012; Waters et al., 2020). In other words, in *Anisocentropus* caddisflies, the lentic water‐adapted species are more restricted in terms of connectivity between populations compared with the lotic water‐adapted species, and so the associated potential for gene flow is also restricted.

However, in general, it is known that species adapted to lentic water conditions have greater dispersal potential and so are associated with wider distribution areas, and also an absence of genetic locality (Hof et al., 2006; Ribera & Vogler, 2000; Yano et al., 2019). Therefore, this is a new finding that the genetic structures of *A. kawamurai* and *A. pallidus* exhibit significantly different patterns from the genetic structures indicated in previous studies. Interestingly, the habitat preference for the lentic water species *A. pallidus* was more localized and specialized than that of typical lentic water‐adapted species, for example, baetid mayfy (Drotz et al., 2012), libellulid dragonflies (Damm et al., 2010), and wetland beetles (Abellán et al., 2009).

In conclusion, we estimated the phylogenetic position of all three Japanese species as being within the cosmopolitan genus *Anisocentropus* and also revealed their dual origin (the western route via land bridge, and the southern route via the Philippine Islands). It was clarified that the two species, *A. kawamurai* and *A. pallidus*, are sister species and constitute a monophyletic clade among the world's *Anisocentropus* caddisflies. We newly suggested that the origin of *Anisocentropus* caddisflies is correctly estimated to be within the South East or East Asian region.

In addition, we revealed a new pattern by means of examining the comparative genetic structures of species inhabiting freshwater environments. Specifically, the contrasting genetic structures of the sister species, *A. kawamurai* and *A. pallidus*, which are distributed in widely overlapped areas throughout the Japanese Islands, were shown to be based on differentiation in their respective adapted environmental preferences (i.e., lentic and lotic waters) and corresponded to the connected status of these habitats. Therefore, we presented evidence that suggests the ecological preferences of a species are an important factor in understanding the evolutionary history of that species.

## CONFLICT OF INTEREST

The authors declare that they have no conflicts of interest.

## AUTHOR CONTRIBUTIONS

**Masaki Takenaka:** Conceptualization (equal); data curation (equal); formal analysis (equal); investigation (lead); methodology (lead); project administration (equal); resources (lead); software (lead); visualization (lead); writing‐original draft (lead); writing‐review & editing (equal). **Saki Shibata** **:** Data curation (equal); formal analysis (equal); investigation (equal). **Tomiko Ito:** Conceptualization (equal); investigation (supporting); writing‐review & editing (supporting). **Noriyoshi Shimura:** Investigation (supporting); writing‐review & editing (supporting). **Koji Tojo** **:** Conceptualization (lead); data curation (supporting); formal analysis (supporting); investigation (equal); methodology (equal); project administration (lead); resources (lead); supervision; visualization (supporting); writing‐review & editing (lead).

## Supporting information

Fig S1

Fig S2

Fig S3

Table S1‐S3

Supplementary Material

## Data Availability

The DNA sequences have been deposited in the public repository of GenBank systems. All methods information is included in this manuscript. All specimens used in this study are stored in the Tojo laboratory of Shinshu University.

## References

[ece37419-bib-0001] Abellán, P. , Millán, A. , & Ribera, I. (2009). Parallel habitat‐driven differences in the phylogeographical structure of two independent lineages of Mediterranean saline water beetles. Molecular Ecology, 18, 3885–3902. 10.1111/j.1365-294X.2009.04319.x 19702753

[ece37419-bib-0002] Arbogast, B. S. , & Kenagy, G. J. (2001). Comparative phylogeography as an integrative approach to historical biogeography. Journal of Biogeography, 28, 819–825. 10.1046/j.1365-2699.2001.00594.x

[ece37419-bib-0003] Arribas, P. , Velasco, J. , Abellán, P. , Sánchez‐Fernández, D. , Andújar, C. , Calosi, P. , Millán, A. , Ribera, I. , & Bilton, D. T. (2012). Dispersal ability rather than ecological tolerance drives differences in range size between lentic and lotic water beetles (Coleoptera: Hydrophilidae). Journal of Biogeography, 39, 984–994. 10.1111/j.1365-2699.2011.02641.x

[ece37419-bib-0004] Bouckaert, R. , Heled, J. , Kühnert, D. , Vaughan, T. , Wu, C.‐H. , Xie, D. , Suchard, M. A. , Rambaut, A. , & Drummond, A. J. (2014). BEAST 2: A software platform for Bayesian evolutionary analysis. PLoS Computational Biology, 10, e1003537. 10.1371/journal.pcbi.1003537 24722319 PMC3985171

[ece37419-bib-0005] Bowler, D. A. , & Benton, T. G. (2005). Causes and consequences of animal dispersal strategies: Relating individual behaviour to spatial dynamics. Biological Reviews, 8, 205–225. 10.1017/S1464793104006645 15921049

[ece37419-bib-0006] Clement, M. , Posada, D. , & Crandall, K. A. (2000). TCS: A computer program to estimate gene genealogies. Molecular Ecology, 9, 1657–1659. 10.1046/j.1365-294x.2000.01020.x 11050560

[ece37419-bib-0007] Damm, S. , Dijkstra, K.‐D.‐B. , & Hadrys, H. (2010). Red drifters and dark residents: The phylogeny and ecology of a Plio‐Pleistocene dragonfly radiation reflects Africa's changing environment (Odonata, Libellulidae, Trithemis). Molecular Phylogenetics and Evolution, 54, 870–882. 10.1016/j.ympev.2009.12.006 20004729

[ece37419-bib-0008] Drotz, M. K. , Savolainen, E. , Saura, A. , & Ståhls, G. (2012). The genetic population structure of lotic and lentic mayflies of the *Baetis vernus* group (Ephemeroptera: Baetidae). Canadian Entomologist, 144, 679–690. 10.4039/tce.2012.69

[ece37419-bib-0009] Ellis, J. S. , Knight, M. E. , Darvill, B. , & Goulson, D. (2006). Extremely low effective population sizes, genetic structuring and reduced genetic diversity in a threatened bumblebee species, *Bombus sylvarum* (Hymenoptera: Apidae). Molecular Ecology, 15, 4375–4386. 10.1111/j.1365-294X.2006.03121.x 17107471

[ece37419-bib-0010] Emms, M. A. , Saenz‐Agudelo, P. , Giles, E. C. , Gatins, R. , Nanninga, G. B. , Scott, A. , Hobbs, J.‐P.‐A. , Frisch, A. J. , Mills, S. C. , Beldade, R. , & Berumen, M. L. (2020). Comparative phylogeography of three host sea anemones in the Indo‐Pacific. Journal of Biogeography, 47, 487–500. 10.1111/jbi.13775

[ece37419-bib-0011] Engel, M. S. , Davis, S. R. , & Prokop, J. (2013). Insect wings: The evolutionary development of nature's first flyers. In A. Minelli (Ed.), Arthropod biology and evolution (pp. 269–298). Springer.

[ece37419-bib-0012] Engel, M. S. , & Grimaldi, D. A. (2004). New light shed on the oldest insect. Nature, 427, 627–630. 10.1038/nature02291 14961119

[ece37419-bib-0013] Excoffier, L. , & Lischer, H. E. L. (2010). Arlequin suite ver 3.5: A new series of programs to perform population genetics analyses under Linux and Windows. Molecular Ecology Resources, 10, 564–567. 10.1111/j.1755-0998.2010.02847.x 21565059

[ece37419-bib-0015] Fu, Y. X. (1997). Statistical tests of neutrality of mutations against population growth, hitchhiking and background selection. Genetics, 147, 915–925. 10.1093/genetics/147.2.915 9335623 PMC1208208

[ece37419-bib-0016] Hanski, I. , & Gyllenberg, M. (1997). Uniting two general patterns in the distribution of species. Science, 275, 397–400. 10.1126/science.275.5298.397 8994039

[ece37419-bib-0017] Hjalmarsson, A. E. , Bergsten, J. , & Monaghan, M. T. (2015). Dispersal is linked to habitat use in 59 species of water beetles (Coleoptera: Adephaga) on Madagascar. Ecography, 38, 732–739. 10.1111/ecog.01138

[ece37419-bib-0075] Holt, B. G. , Lessard, J. P. , Borregaard, M. K. , Fritz, S. A. , Araújo, M. B. , Dimitrov, D. , Fabre, P. H. , Graham, C. H. , Graves, G. R. , Jønsson, K. A. , Nogués‐Bravo, D. , Wang, Z. , Whittaker, R. J. , Fjeldså, J. , & Rahbek, C. (2013). An update of Wallace’s Zoogeographic regions of the World. Science, 339(6115), 74–78. 10.1126/science.1228282 23258408

[ece37419-bib-0018] Hof, C. , Brändle, M. , & Brandl, R. (2006). Lentic odonates have larger and more northern ranges than lotic species. Journal of Biogeography, 33, 63–70. 10.1111/j.1365-2699.2005.01358.x

[ece37419-bib-0019] Hof, C. , Brändle, M. , Dehling, D. M. , Munguía, M. , Brandl, R. , Araújo, M. B. , & Rahbek, C. (2012). Habitat stability affects dispersal and the ability to track climate change. Biology Letters, 8, 639–643. 10.1098/rsbl.2012.0023 22378743 PMC3391453

[ece37419-bib-0020] Hughes, J. M. , Huey, J. A. , & Schmidt, D. J. (2013). Is realised connectivity among populations of aquatic fauna predictable from potential connectivity? Freshwater Biology, 58, 951–966. 10.1111/fwb.12099

[ece37419-bib-0021] Ikeda, H. , Nishikawa, M. , & Sota, T. (2012). Loss of flight promotes beetle diversification. Nature Communications, 3, 1–8. 10.1038/ncomms1659 PMC3272566 22337126

[ece37419-bib-0022] Ito, T. (2016). Biology of *Anisocentropus pallidus* (Martynov) (Trichoptera, Calamoceratidae): Laboratory and field observations. Zoosymposia, 10, 214–223. 10.11646/zoosymposia.10.1.19

[ece37419-bib-0023] Ito, T. (2017). A checklist of caddisflies (Trichoptera) of the Ryukyu Archipelago, southwestern Japan. Biology of Inland Waters, 32, 87–105 [In Japanese with English abstract].

[ece37419-bib-0024] Ito, T. , Hayashi, Y. , & Shimura, N. (2012). The genus *Anisocentropus* McLachlan (Trichoptera, Calamoceratidae) in Japan. Zootaxa, 3157, 1–17. 10.11646/zootaxa.3157.1.1

[ece37419-bib-0025] Kato, Y. , Morii, Y. , & Tojo, K. (2013). Molecular phylogeographic analysis of East Asian cryptoperlan stoneflies (Insecta: Plecoptera, Peltoperlidae). Limnology, 14, 179–194. 10.1007/s10201-012-0395-3

[ece37419-bib-0026] Katoh, K. , & Standley, D. M. (2013). MAFFT Multiple sequence alignment software version 7: Improvements in performance and usability. Molecular Biology and Evolution, 30, 772–780. 10.1093/molbev/mst010 23329690 PMC3603318

[ece37419-bib-0027] Kellermann, V. , Van Heerwaarden, B. , Sgrò, C. M. , & Hoffmann, A. A. (2009). Fundamental evolutionary limits in ecological traits drive *Drosophila* species distributions. Science, 325, 1244–1246. 10.1126/science.1175443 19729654

[ece37419-bib-0028] Kim, C. G. , Zhou, H. Z. , Imura, Y. , Tominaga, O. , Su, Z. H. , & Osawa, S. (2000). Pattern of morphological diversification in the *Leptocarabus* ground beetles (Coleoptera: Carabidae) as deduced from mitochondrial ND5 gene and nuclear 28S rDNA sequences. Molecular Biology and Evolution, 17, 137–145. 10.1093/oxfordjournals.molbev.a026226 10666713

[ece37419-bib-0029] Koizumi, Y. , Ota, H. , & Hikida, T. (2014). Phylogeography of the two smooth skinks, *Scincella boettgeri* and *S. formosensis* (Squamata: Scincidae) in the southern Ryukyus and Taiwan, as inferred from variation in mitochondrial cytochrome b sequences. Zoological Science, 31, 228–236. 10.2108/zs130180 24694225

[ece37419-bib-0030] Larson, D. J. (1997). Habitat and community patterns of tropical Australian *Hydradephagan* water beetles (Coleoptera: Dytiscidae, Gyrinidae, Noteridae). Australian Journal of Entomology, 36, 269–285. 10.1111/j.1440-6055.1997.tb01469.x

[ece37419-bib-0031] Leaché, A. D. , Oaks, J. R. , Ofori‐Boateng, C. , & Fujita, M. K. (2020). Comparative phylogeography of West African amphibians and reptiles. Evolution, 74, 716–724. 10.1111/evo.13941 32067219

[ece37419-bib-0032] Leigh, J. W. , & Bryant, D. (2015). popart: Full‐feature software for haplotype network construction. Methods in Ecology and Evolution, 6, 1110–1116. 10.1111/2041-210X.12410

[ece37419-bib-0033] Leys, M. , Keller, I. , Räsänen, K. , Gattolliat, J. L. , & Robinson, C. T. (2016). Distribution and population genetic variation of cryptic species of the Alpine mayfly *Baetis alpinus* (Ephemeroptera: Baetidae) in the Central Alps. BMC Evolutionary Biology, 16, 1–15. 10.1186/s12862-016-0643-y 27068234 PMC4828801

[ece37419-bib-0034] MacArthur, R. H. , & Wilson, E. O. (2001). The theory of island biogeography. New Jersey: Princeton University Press.

[ece37419-bib-0035] Martin, A. K. (2011). Double saloon door tectonics in the Japan Sea, Fossa magna, and the Japanese Island arc. Tectonophysics, 498, 45–65.

[ece37419-bib-0036] Misof, B. , Liu, S. , Meusemann, K. , Peters, R. S. , Donath, A. , Mayer, C. , Frandsen, P. B. , Ware, J. , Flouri, T. , Beutel, R. G. , Niehuis, O. , Petersen, M. , Izquierdo‐Carrasco, F. , Wappler, T. , Rust, J. , Aberer, A. J. , Aspock, U. , Aspock, H. , Bartel, D. , … Zhou, X. (2014). Phylogenomics resolves the timing and pattern of insect evolution. Science, 346, 763–767. 10.1126/science.1257570 25378627

[ece37419-bib-0037] Morse, J. C. (Ed.) (2020). Trichoptera world checklist. Retrieved from http://entweb.clemson.edu/database/trichopt/index.htm

[ece37419-bib-0038] Motokawa, M. , & Kajihara, H. (2017). Species diversity of animals in Japan. Tokyo: Springer Japan.

[ece37419-bib-0039] Múrria, C. , & Hughes, J. M. (2008). Cyclic habitat displacements during Pleistocene glaciations have induced independent evolution of *Tasimia palpata* populations (Trichoptera: Tasimiidae) in isolated subtropical rain forest patches. Journal of Biogeography, 35, 1727–1737. 10.1111/j.1365-2699.2008.01918.x

[ece37419-bib-0040] Nei, M. (1987). Molecular evolutionary genetics. New York: Columbia University Press.

[ece37419-bib-0041] Nei, M. , & Li, W. H. (1979). Mathematical model for studying genetic variation in terms of restriction endonucleases. Proceedings of the National Academy of Sciences of the United States of America, 76, 5269–5273. 10.1073/pnas.76.10.5269 291943 PMC413122

[ece37419-bib-0042] Nozaki, T. , Saito, R. , Nishimura, N. , Hsu, L. P. , & Tojo, K. (2016). Larvae and females of two *Stenopsyche* species in Taiwan with redescription of the male of *S. formosana* (Insecta: Trichoptera). Zootaxa, 4121, 485–494. 10.11646/zootaxa.4121.4.8 27395237

[ece37419-bib-0044] Okamiya, H. , Sugawara, H. , Nagano, M. , & Poyarkov, N. A. (2018). An integrative taxonomic analysis reveals a new species of lotic *Hynobius* salamander from Japan. PeerJ, 6, e5084. 10.7717/peerj.5084 29942708 PMC6015758

[ece37419-bib-0076] Okamoto, S. , & Tojo, K. (2021). Distribution patterns and niche segregation of three closely related Japanese ephemerid mayflies: a re‐examination of each species’ habitat from “megadata” held in the “National Census on River Environments”. Limnology. 10.1007/s10201-021-00654-2

[ece37419-bib-0045] Osozawa, S. , Sato, F. , & Wakabayashi, J. (2017). Quaternary vicariance of lotic *Coeliccia* in the Ryukyu‐Taiwan Islands contrasted with lentic *Copera* . Journal of Heredity, 108, 280–287. 10.1093/jhered/esx007 28164229

[ece37419-bib-0046] Otofuji, Y. I. , Matsuda, T. , & Nohda, S. (1985). Opening mode of the Japan Sea inferred from the palaeomagnetism of the Japan Arc. Nature, 317, 603–604. 10.1038/317603a0

[ece37419-bib-0048] Rambaut, A. (2009). FigTree, Version 1.3.1. Retrieved from http://tree.bio.ed.ac.uk/software/figtree/

[ece37419-bib-0049] Rambaut, A. , Suchard, M. A. , Xie, D. , & Drummond, A. J. (2014). Tracer, version 1.6, MCMC trace analysis package. Retrieved from http://tree.bio.ed.ac.uk/software/tracer/

[ece37419-bib-0050] Ribera, I. , Bilton, D. T. , & Vogler, A. P. (2003). Mitochondrial DNA phylogeography and population history of *Meladema* diving beetles on the Atlantic Islands and in the Mediterranean basin (Coleoptera, Dytiscidae). Molecular Ecology, 12, 153–167. 10.1046/j.1365-294X.2003.01711.x 12492885

[ece37419-bib-0051] Ribera, I. , & Vogler, A. P. (2000). Habitat type as a determinant of species range sizes: The example of lotic‐lentic differences in aquatic Coleoptera. Biological Journal of the Linnean Society, 71, 33–52. 10.1111/j.1095-8312.2000.tb01240.x

[ece37419-bib-0052] Rozas, J. , Sánchez‐DelBarrio, J. C. , Messeguer, X. , & Rozas, R. (2003). DnaSP, DNA polymorphism analyses by the coalescent and other methods. Bioinformatics, 19, 2496–2497. 10.1093/bioinformatics/btg359 14668244

[ece37419-bib-0053] Saito, R. , Kato, S. , Kuranishi, R. B. , Nozaki, T. , Fujino, T. , & Tojo, K. (2018). Phylogeographic analyses of the *Stenopsyche* caddisflies (Trichoptera: Stenopsychidae) of the Asian Region. Freshwater Science, 37, 562–572. 10.1086/699364

[ece37419-bib-0054] Saito, R. , & Tojo, K. (2016a). Complex geographic‐and habitat‐based niche partitioning of an East Asian habitat generalist mayfly *Isonychia japonica* (Ephemeroptera: Isonychiidae) with reference to differences in genetic structure. Freshwater Science, 35, 712–723. 10.1086/686564

[ece37419-bib-0055] Saito, R. , & Tojo, K. (2016b). Comparing spatial patterns of population density, biomass, and genetic diversity patterns of the habitat generalist mayfly *Isonychia japonica* Ulmer (Ephemeroptera: Isonychiidae) in the Chikuma‐Shinano River basin. Freshwater Science, 35, 724–737. 10.1086/686537

[ece37419-bib-0056] Schwarz, G. (1978). Estimating the dimension of a model. Annals of Statistics, 6, 461–464. 10.1214/aos/1176344136

[ece37419-bib-0057] Sekiné, K. , Hayashi, F. , & Tojo, K. (2013). Phylogeography of the East Asian polymitarcyid mayfly genus *Ephoron* (Ephemeroptera: Polymitarcyidae): A comparative analysis of molecular and ecological characteristics. Biological Journal of the Linnean Society, 109, 181–202. 10.1111/bij.12033

[ece37419-bib-0058] Sekiné, K. , Tojo, K. , & Bae, Y. J. (2015). Distribution and genetic characteristics of *Ephoron shigae* (Ephemeroptera: Polymitarcyidae) in Korea. Entomological Research, 45, 150–157. 10.1111/1748-5967.12107

[ece37419-bib-0059] Su, Z. H. , Ohama, T. , Okada, T. S. , Nakamura, K. , Ishikawa, R. , & Osawa, S. (1996). Geography‐linked phylogeny of the Damaster ground beetles inferred from mitochondrial ND5 gene sequences. Journal of Molecular Evolution, 42, 130–134. 10.1007/BF02198838 8995063

[ece37419-bib-0060] Suwannarat, N. , Malicky, H. , & Laudee, P. (2018). Two new species of Caddisflies (Trichoptera: Insecta) from Lower‐Hill Evergreen Forests of Southern Thailand. Zootaxa, 4524, 496–500. 10.11646/zootaxa.4524.4.7 30486109

[ece37419-bib-0061] Suzuki, T. , Kitano, T. , & Tojo, K. (2014). Contrasting genetic structure of closely related giant water bugs: Phylogeography of *Appasus japonicus* and *Appasus major* (Insecta: Heteroptera, Belostomatidae). Molecular Phylogenetics and Evolution, 72, 7–16. 10.1016/j.ympev.2013.12.008 24398367

[ece37419-bib-0062] Tajima, F. (1989). Statistical method for testing the neutral mutation hypothesis by DNA polymorphism. Genetics, 123, 585–595. 10.1093/genetics/123.3.585 2513255 PMC1203831

[ece37419-bib-0063] Takenaka, M. , Shibata, S. , Shimura, N. , Ito, T. , & Tojo, K. (2019). Update on distribution area information of three species of *Anisocentropus* caddisflies inhabiting the Japanese Islands (Trichoptera, Calamoceratidae). New Entomologist, 67, 13–20.

[ece37419-bib-0064] Takenaka, M. , & Tojo, K. (2019). Ancient origin of a dipteromimid mayfly family endemic to the Japanese Islands and its genetic differentiation across tectonic faults. Biological Journal of the Linnean Society, 126, 555–573. 10.1093/biolinnean/bly192

[ece37419-bib-0065] Takenaka, M. , Tokiwa, T. , & Tojo, K. (2019). Concordance between molecular biogeography of *Dipteromimus tipuliformis* and geological history in the local fine scale (Ephemeroptera, Dipteromimidae). Molecular Phylogenetics and Evolution, 139, 106547. 10.1016/j.ympev.2019.106547 31260742

[ece37419-bib-0066] Tamura, K. , Stecher, G. , Peterson, D. , Filipski, A. , & Kumar, S. (2013). MEGA6: Molecular Evolutionary Genetics Analysis version 6.0. Molecular Biology and Evolution, 30, 2725–2729. 10.1093/molbev/mst197 24132122 PMC3840312

[ece37419-bib-0067] Tanabe, A. S. (2007). Kakusan: A computer program to automate the selection of a nucleotide substitution model and the configuration of a mixed model on multilocus data. Molecular Ecology Notes, 7, 962–964. 10.1111/j.1471-8286.2007.01807.x

[ece37419-bib-0068] Thomas, J. A. , Frandsen, P. B. , Prendini, E. , Zhou, X. , & Holzenthal, R. W. (2020). A multigene phylogeny and timeline for Trichoptera (Insecta). Systematic Entomology, 45, 670–686. 10.1111/syen.12422

[ece37419-bib-0069] Tojo, K. , Sekiné, K. , Takenaka, M. , Isaka, Y. , Komaki, S. , Suzuki, T. , & Schoville, S. D. (2017). Species diversity of insects in Japan: Their origins and diversification processes. Entomological Science, 20, 357–380. 10.1111/ens.12261

[ece37419-bib-0070] Tseng, H. Y. , Huang, W. S. , Jeng, M. L. , Villanueva, R. J. T. , Nuñeza, O. M. , & Lin, C. P. (2018). Complex inter‐island colonization and peripatric founder speciation promote diversification of flightless *Pachyrhynchus weevils* in the Taiwan‐Luzon volcanic belt. Journal of Biogeography, 45, 89–100. 10.1111/jbi.13110

[ece37419-bib-0071] Waters, J. M. , Emerson, B. C. , Arribas, P. , & McCulloch, G. A. (2020). Dispersal reduction: Causes, genomic mechanisms, and evolutionary consequences. Trends in Ecology & Evolution, 35, 512–522. 10.1016/j.tree.2020.01.012 32396818

[ece37419-bib-0072] Yano, K. , Takenaka, M. , & Tojo, K. (2019). Genealogical position of Japanese populations of the globally distributed mayfly *Cloeon dipterum* and related species (Ephemeroptera, Baetidae): A molecular phylogeographic analysis. Zoological Science, 36, 479–489. 10.2108/zs190049 31833319

[ece37419-bib-0073] Yu, Y. , Blair, C. , & He, X. (2020). RASP 4: Ancestral state reconstruction tool for multiple genes and characters. Molecular Biology and Evolution, 37, 604–606. 10.1093/molbev/msz257 31670774

[ece37419-bib-0074] Zhou, X. , Frandsen, P. B. , Holzenthal, R. W. , Beet, C. R. , Bennett, K. R. , Blahnik, R. J. , Bonada, N. , Cartwright, D. , Chuluunbat, S. , Cocks, G. V. , Collins, G. E. , deWaard, J. , Dean, J. , Flint, O. S. , Hausmann, A. , Hendrich, L. , Hess, M. , Hogg, I. D. , Kondratieff, B. C. , … Kjer, K. M. (2016). The Trichoptera barcode initiative: A strategy for generating a species‐level Tree of Life. Philosophical Transactions of the Royal Society B: Biological Sciences, 371, 20160025. 10.1098/rstb.2016.0025 PMC4971193 27481793

