# Supplementary material for: Phylogeography of the northernmost distributed Anisocentropus caddisflies and their comparative genetic structures based on habitat preferences
Source: Ecol Evol. 2021 Mar 30;11(9):4957–71. doi: 10.1002/ece3.7419 (PMC8093727; doi:10.1002/ece3.7419)
Supplement: Supplementary file 4 — Table S1‐S3 [file ECE3-11-4957-s002.docx]

| Table S1. List of specimens of *Anisocentropus* caddisflies examined in this study, sequence types, and the GenBank accession numbers | | | | | | | | | |
| --- | --- | --- | --- | --- | --- | --- | --- | --- | --- |
|  | Sampling sites | |  |  |  | mtDNA COI | | nDNA histone H3 | |
|  | Collecting site's No. and location name | | Country | Latitude (N) | Longitude (E) | Haplotype(s) | GenBank acces. No. | Haplotype(s) | GenBank acces. No. |
| ***Anisocentropus kawamurai*** | | |  |  |  |  |  |  |  |
|  | 1 | Koshunai, Bibai, Hokkaido | Japan | 43.28333 | 141.85000 | Ak_43 | LC605202 | Ak_1 | LC605454 |
|  |  |  |  |  |  | Ak_43 | LC605203 |  |  |
|  |  |  |  |  |  | Ak_43 | LC605204 |  |  |
|  | 2 | Komasato, Chitose, Hokkaido | Japan | 42.80000 | 141.71667 | Ak_41 | LC605205 |  |  |
|  |  |  |  |  |  | Ak_41 | LC605206 |  |  |
|  |  |  |  |  |  | Ak_41 | LC605207 | - |  |
|  |  |  |  |  |  | Ak_41 | LC605208 | Ak_1 | LC605455 |
|  |  |  |  |  |  | Ak_41 | LC605209 |  |  |
|  | 3 | Uenae, Tomakomai, Hokkaido | Japan | 42.72361 | 141.71056 | Ak_41 | LC605210 | - |  |
|  | 4 | Hitsujigaoka, Sapporo, Hokkaido | Japan | 43.01054 | 141.41113 | Ak_43 | LC605211 | - |  |
|  | 5 | Banjiri, Eniwa, Hokkaido | Japan | 42.85000 | 141.38333 | Ak_43 | LC605212 | Ak_1 | LC605456 |
|  |  |  |  |  |  | Ak_43 | LC605213 | Ak_1 | LC605457 |
|  | 6 | Onumacho, Nanae, Hokkaido | Japan | 41.98333 | 140.66667 | Ak_44 | LC605214 |  |  |
|  | 7 | Kawai, Miyako, Iwate | Japan | 39.60179 | 141.67924 | Ak_38 | LC605215 |  |  |
|  |  |  |  |  |  | Ak_31 | LC605216 |  |  |
|  | 8 | Towa, Tome, Miyagi | Japan | 38.75320 | 141.38577 | Ak_2 | LC605217 |  |  |
|  |  |  |  |  |  | Ak_39 | LC605218 | - |  |
|  |  |  |  |  |  | Ak_13 | LC605219 | Ak_1 | LC605458 |
|  | 9 | Kitakami, Ishinomaki, Miyagi | Japan | 38.57991 | 141.38199 | Ak_42 | LC605220 | Ak_1 | LC605459 |
|  |  |  |  |  |  | Ak_2 | LC605221 | - |  |
|  |  |  |  |  |  | Ak_40 | LC605222 |  |  |
|  | 10 | Aramaki, Sendai, Miyagi | Japan | 38.24800 | 140.83440 | Ak_2 | LC605223 |  |  |
|  |  |  |  |  |  | Ak_19 | LC605224 |  |  |
|  |  |  |  |  |  | Ak_12 | LC605225 |  |  |
|  | 11 | Kashiwagiyama, Shichigajuku, Miyagi | Japan | 38.02870 | 140.47823 | Ak_2 | LC605226 | Ak_1 | LC605460 |
|  | 12 | Yoshima, Iwaki, Fukushima | Japan | 37.06358 | 140.85726 | Ak_19 | LC605227 | Ak_2 | LC605461 |
|  | 13 | Kitayamamoto, Tanagura, Fukushima | Japan | 36.98816 | 140.31711 | Ak_5 | LC605228 | Ak_1 | LC605462 |
|  |  |  |  |  |  | Ak_21 | LC605229 | Ak_1 | LC605463 |
|  | 14 | Odakura, Nishigo, Fukushima | Japan | 37.15246 | 140.08420 | Ak_12 | LC605230 | Ak_1 | LC605464 |
|  | 15 | Kamioba, Mashiko, Tochigi | Japan | 36.44731 | 140.14086 | Ak_2 | LC605231 | - |  |
|  | 16 | Hinatawada, Ome, Tokyo | Japan | 35.78306 | 139.23111 | Ak_34 | LC605232 | Ak_1 | LC605465 |
|  | 17 | Oguno, Hinode, Tokyo | Japan | 35.77750 | 139.19083 | Ak_2 | LC605233 | Ak_1 | LC605466 |
|  | 18 | Yozawa, Akiruno, Tokyo | Japan | 35.75139 | 139.18222 | Ak_2 | LC605234 | - |  |
|  |  |  |  |  |  | Ak_2 | LC605235 |  |  |
|  |  |  |  |  |  | Ak_4 | LC605236 |  |  |
|  | 19 | Sano, Izu, Shizuoka | Japan | 34.93878 | 138.94652 | Ak_18 | LC605237 | Ak_1 | LC605467 |
|  |  |  |  |  |  | Ak_2 | LC605238 |  |  |
|  |  |  |  |  |  | Ak_2 | LC605239 |  |  |
|  | 20 | Haruno, Hamamatsu, Shizuoka | Japan | 35.01994 | 137.92693 | Ak_2 | LC605240 | Ak_1 | LC605468 |
|  | 21 | Fukushi, Nambu, Yamanashi | Japan | 35.20051 | 138.43933 | Ak_2 | LC605241 |  |  |
|  |  |  |  |  |  | Ak_2 | LC605242 |  |  |
|  |  |  |  |  |  | Ak_2 | LC605243 | Ak_1 | LC605469 |
|  | 22 | Ohinata, Sakuho, Nagano | Japan | 36.12946 | 138.57826 | Ak_2 | LC605244 |  |  |
|  |  |  |  |  |  | Ak_2 | LC605245 | - |  |
|  | 23 | Nakatsunako, Omachi, Nagano | Japan | 36.59737 | 137.84364 | Ak_6 | LC605246 |  |  |
|  |  |  |  |  |  | Ak_6 | LC605247 |  |  |
|  | 24 | Kizakiko, Omachi, Nagano | Japan | 36.55586 | 137.83357 | Ak_2 | LC605248 |  |  |
|  |  |  |  |  |  | Ak_6 | LC605249 | Ak_1 | LC605470 |
|  | 25 | Shimoyoshida, Shinshiro, Aichi | Japan | 34.95028 | 137.64539 | Ak_3 | LC605250 |  |  |
|  | 26 | Tsukude, Shinshiro, Aichi | Japan | 34.95039 | 137.45714 | Ak_2 | LC605251 | Ak_1 | LC605471 |
|  |  |  |  |  |  | Ak_2 | LC605252 |  |  |
|  |  |  |  |  |  | Ak_2 | LC605253 |  |  |
|  | 27 | Goyu, Toyokawa, Aichi | Japan | 34.84006 | 137.31147 | Ak_3 | LC605254 | Ak_1 | LC605472 |
|  |  |  |  |  |  | Ak_3 | LC605255 | - |  |
|  |  |  |  |  |  | Ak_3 | LC605256 |  |  |
|  | 28 | Nakaoku, Kawakami, Nara | Japan | 34.32258 | 136.03970 | Ak_2 | LC605257 |  |  |
|  | 29 | Kawai, Kamikitayama, Nara | Japan | 34.15723 | 135.97515 | Ak_2 | LC605258 |  |  |
|  |  |  |  |  |  | Ak_8 | LC605259 |  |  |
|  |  |  |  |  |  | Ak_30 | LC605260 | Ak_1 | LC605473 |
|  | 30 | Kumogahata, Kyoto, Kyoto | Japan | 35.10972 | 135.72444 | Ak_15 | LC605261 | Ak_1 | LC605474 |
|  |  |  |  |  |  | Ak_15 | LC605262 | - |  |
|  |  |  |  |  |  | AK_18 | LC605263 | - |  |
|  | 31 | Ayukawa, Tanabe, Wakayama | Japan | 33.72672 | 135.49660 | Ak_17 | LC605264 |  |  |
|  | 32 | Kotsumi, Kaizuka, Osaka | Japan | 34.37350 | 135.40544 | Ak_2 | LC605265 |  |  |
|  | 33 | Wada, Asago, Hyogo | Japan | 35.34432 | 134.89872 | Ak_7 | LC605266 |  |  |
|  |  |  |  |  |  | Ak_45 | LC605267 | Ak_1 | LC605475 |
|  | 34 | Yumesaki, Himeji, Hyogo | Japan | 34.91482 | 134.64244 | Ak_2 | LC605268 | Ak_1 | LC605476 |
|  |  |  |  |  |  | Ak_2 | LC605269 |  |  |
|  |  |  |  |  |  | Ak_16 | LC605270 |  |  |
|  |  |  |  |  |  | Ak_2 | LC605271 |  |  |
|  | 35 | Donari, Awa, Tokushima | Japan | 34.16840 | 134.32475 | Ak_27 | LC605272 |  |  |
|  |  |  |  |  |  | Ak_2 | LC605273 |  |  |
|  |  |  |  |  |  | Ak_14 | LC605274 | Ak_1 | LC605477 |
|  | 36 | Shionoe, Takamatsu, Kagawa | Japan | 34.13858 | 134.07731 | Ak_3 | LC605275 |  |  |
|  |  |  |  |  |  | Ak_2 | LC605276 | - |  |
|  | 37 | Jinryo, Kamiyama, Tokushima | Japan | 33.95644 | 134.36653 | Ak_18 | LC605277 | Ak_1 | LC605478 |
|  |  |  |  |  |  | Ak_2 | LC605278 |  |  |
|  |  |  |  |  |  | Ak_2 | LC605279 |  |  |
|  | 38 | Yamashiro, Miyoshi, Tokushima | Japan | 33.89821 | 133.70662 | Ak_36 | LC605280 | Ak_1 | LC605479 |
|  | 39 | Inokuchi, Aki, Kochi | Japan | 33.53412 | 133.91498 | Ak_2 | LC605281 | - |  |
|  |  |  |  |  |  | Ak_2 | LC605282 | - |  |
|  | 40 | Tosayamada, Kami, Kochi | Japan | 33.62100 | 133.73203 | Ak_2 | LC605283 |  |  |
|  |  |  |  |  |  | Ak_2 | LC605284 |  |  |
|  | 41 | Enomura, Shimanto, Kochi | Japan | 32.95753 | 132.86651 | Ak_2 | LC605285 | Ak_1 | LC605480 |
|  |  |  |  |  |  | Ak_2 | LC605286 | - |  |
|  | 42 | Kaidani, Okayama, Okayama | Japan | 34.71939 | 133.90226 | Ak_45 | LC605287 |  |  |
|  |  |  |  |  |  | Ak_47 | LC605288 |  |  |
|  | 43 | Otani, Sera, Hiroshima | Japan | 34.63015 | 133.12557 | Ak_2 | LC605289 | Ak_1 | LC605481 |
|  |  |  |  |  |  | Ak_2 | LC605290 |  |  |
|  | 44 | Akitsu, Higashi-hiroshima, Hiroshima | Japan | 34.35323 | 132.81335 | Ak_54 | LC605291 |  |  |
|  |  |  |  |  |  | Ak_9 | LC605292 |  |  |
|  |  |  |  |  |  | Ak_9 | LC605293 | Ak_1 | LC605482 |
|  |  |  |  |  |  | Ak_2 | LC605294 |  |  |
|  | 45 | Kariyagata, Kita-hiroshima, Hiroshima | Japan | 34.76579 | 132.24837 | Ak_22 | LC605295 | Ak_1 | LC605483 |
|  |  |  |  |  |  | Ak_2 | LC605296 |  |  |
|  | 46 | Sambe, Oda, Shimane | Japan | 35.13146 | 132.59265 | Ak_2 | LC605297 |  |  |
|  |  |  |  |  |  | Ak_45 | LC605298 | Ak_1 | LC605484 |
|  |  |  |  |  |  | Ak_2 | LC605299 |  |  |
|  | 47 | Tennou, Iwakuni, Yamaguchi | Japan | 34.18780 | 132.07217 | Ak_2 | LC605300 |  |  |
|  |  |  |  |  |  | Ak_33 | LC605301 |  |  |
|  |  |  |  |  |  | Ak_45 | LC605302 | Ak_1 | LC605485 |
|  | 48 | Mikawa, Iwakuni, Yamaguchi | Japan | 34.22048 | 131.97910 | Ak_2 | LC605303 | Ak_1 | LC605486 |
|  |  |  |  |  |  | Ak_10 | LC605304 | - |  |
|  |  |  |  |  |  | Ak_33 | LC605305 |  |  |
|  | 49 | Suyama, Shunan, Yamaguchi | Japan | 34.23761 | 131.76224 | Ak_2 | LC605306 |  |  |
|  | 50 | Tokujifukadani, Yamaguchi, Yamaguchi | Japan | 34.21533 | 131.70253 | Ak_11 | LC605307 |  |  |
|  |  |  |  |  |  | Ak_35 | LC605308 | Ak_1 | LC605487 |
|  |  |  |  |  |  | Ak_2 | LC605309 | - |  |
|  | 51 | Nagu, Okinoshi, Shimane | Japan | 36.23963 | 133.23294 | Ak_29 | LC605310 |  |  |
|  |  |  |  |  |  | Ak_37 | LC605311 |  |  |
|  | 52 | Ogura, Kitakyushu, Fukuoka | Japan | 33.79043 | 130.81761 | Ak_2 | LC605312 | Ak_1 | LC605488 |
|  |  |  |  |  |  | Ak_2 | LC605313 |  |  |
|  |  |  |  |  |  | Ak_2 | LC605314 |  |  |
|  | 53 | Ino, Hisayama, Fukuoka | Japan | 33.67181 | 130.52018 | Ak_45 | LC605315 | Ak_1 | LC605489 |
|  |  |  |  |  |  | Ak_22 | LC605316 |  |  |
|  |  |  |  |  |  | Ak_53 | LC605317 |  |  |
|  | 54 | Imaichi, Oita, Oita | Japan | 33.09948 | 131.45916 | Ak_2 | LC605318 | Ak_1 | LC605490 |
|  |  |  |  |  |  | Ak_44 | LC605319 |  |  |
|  |  |  |  |  |  | Ak_44 | LC605320 |  |  |
|  | 55 | Innai, Usa, Oita | Japan | 33.40082 | 131.31971 | Ak_22 | LC605321 | Ak_1 | LC605491 |
|  |  |  |  |  |  | Ak_22 | LC605322 |  |  |
|  |  |  |  |  |  | Ak_25 | LC605323 |  |  |
|  | 56 | Yamakuni, Nakatsu, Oita | Japan | 33.49742 | 130.97674 | Ak_23 | LC605324 |  |  |
|  |  |  |  |  |  | Ak_28 | LC605325 | Ak_1 | LC605492 |
|  |  |  |  |  |  | Ak_2 | LC605326 |  |  |
|  | 57 | Kitagokurogi, Misato, Miyazaki | Japan | 32.52100 | 131.50937 | Ak_24 | LC605327 | Ak_1 | LC605493 |
|  | 58 | Kitamata, Aya, Miyazaki | Japan | 32.03100 | 131.20500 | Ak_51 | LC605328 | Ak_1 | LC605494 |
|  |  |  |  |  |  | Ak_22 | LC605329 | - |  |
|  |  |  |  |  |  | Ak_45 | LC605330 | - |  |
|  |  |  |  |  |  | Ak_51 | LC605331 | - |  |
|  |  |  |  |  |  | Ak_45 | LC605332 | - |  |
|  |  |  |  |  |  | Ak_46 | LC605333 |  |  |
|  |  |  |  |  |  | Ak_45 | LC605334 |  |  |
|  |  |  |  |  |  | Ak_45 | LC605335 |  |  |
|  | 59 | Nishisata, Kagoshima, Kagoshima | Japan | 31.73381 | 130.52763 | Ak_49 | LC605336 |  |  |
|  |  |  |  |  |  | Ak_50 | LC605337 |  |  |
|  | 60 | Chiran, Minami-kyushu, Kagoshima | Japan | 31.32789 | 130.46545 | Ak_45 | LC605338 |  |  |
|  |  |  |  |  |  | Ak_26 | LC605339 |  |  |
|  | 61 | Fukiage, Hioki, Kagoshima | Japan | 31.52929 | 130.40253 | Ak_14 | LC605340 |  |  |
|  |  |  |  |  |  | Ak_45 | LC605341 |  |  |
|  | 62 | Kamitsushima, Tsushima, Nagasaki | Japan | 34.68286 | 129.44952 | Ak_1 | LC605342 | Ak_1 | LC605495 |
|  | 63 | Toyotama, Tsushima, Nagasaki | Japan | 34.40627 | 129.32928 | Ak_2 | LC605343 | - |  |
|  | 64 | Mitsushima, Tsushima, Nagasaki | Japan | 34.26929 | 129.27080 | Ak_2 | LC605344 | - |  |
|  | 65 | Izuhara, Tsushima, Nagasaki | Japan | 34.12956 | 129.23227 | Ak_2 | LC605345 | - |  |
|  | 66 | Kishiku, Goto, Nagasaki | Japan | 32.71000 | 128.67556 | AK_52 | LC605346 | Ak_1 | LC605496 |
|  |  |  |  |  |  | AK_52 | LC605347 | - |  |
|  | 67 | Sumiyoshi, Nishino-omote, Kagoshima | Japan | 30.69220 | 130.96986 | Ak_45 | LC605348 | - |  |
|  |  |  |  |  |  | Ak_45 | LC605349 |  |  |
|  | 68 | Sakai, Nakatane, Kagoshima | Japan | 30.46462 | 130.92128 | Ak_45 | LC605350 |  |  |
|  |  |  |  |  |  | Ak_45 | LC605351 |  |  |
|  | 69 | Nakanoshima, Toshima, Kagoshima | Japan | 29.85266 | 129.88573 | Ak_48 | LC605352 | Ak_1 | LC605497 |
|  |  |  |  |  |  | Ak_45 | LC605353 | Ak_1 | LC605498 |
|  | 70 | Yonaguni, Yonaguni, Okinawa | Japan | 24.44878 | 122.96802 | Ak_65 | LC605354 | Ak_1 | LC605499 |
|  | 71 | Jangsan, Ganghyeon, Gangwon-do | Korea | 38.12917 | 128.58250 | Ak_68 | LC605355 | - |  |
|  |  |  |  |  |  | Ak_67 | LC605356 |  |  |
|  |  |  |  |  |  | Ak_68 | LC605357 |  |  |
|  | 72 | Icheon-ri, Samcheog, Gangwon-do | Korea | 37.20173 | 129.29384 | Ak_59 | LC605358 | Ak_1 | LC605500 |
|  |  |  |  |  |  | Ak_56 | LC605359 | Ak_1 | LC605501 |
|  |  |  |  |  |  | Ak_69 | LC605360 | Ak_1 | LC605502 |
|  | 73 | Samyang-ri, Sannae, Gyeongsangnam-do | Korea | 35.58698 | 128.99698 | Ak_55 | LC605361 | Ak_1 | LC605503 |
|  |  |  |  |  |  | Ak_57 | LC605362 | Ak_3 | LC605504 |
|  | 74 | Gucheon, Danjang, Gyeongsangnam-do | Korea | 35.53264 | 128.96125 | Ak_55 | LC605363 | Ak_3 | LC605505 |
|  |  |  |  |  |  | Ak_56 | LC605364 | Ak_3 | LC605506 |
|  | 75 | Damseong, Sancheong, Gyeonsangnam-do | Korea | 35.33802 | 127.89852 | Ak_55 | LC605365 | Ak_1 | LC605507 |
|  |  |  |  |  |  | Ak_55 | LC605366 | Ak_1 | LC605508 |
|  |  |  |  |  |  | Ak_56 | LC605367 | Ak_1 | LC605509 |
|  | 76 | Sayang, Unam, Jeollabuk-do | Korea | 35.65444 | 127.16028 | Ak_60 | LC605368 | - |  |
|  |  |  |  |  |  | Ak_66 | LC605369 |  |  |
|  |  |  |  |  |  | Ak_66 | LC605370 |  |  |
|  | 77 | Naejangsang, Jeongeup, Jeollabuk-do | Korea | 35.48732 | 126.90824 | Ak_66 | LC605371 | Ak_1 | LC605510 |
|  |  |  |  |  |  | Ak_58 | LC605372 | Ak_1 | LC605511 |
|  |  |  |  |  |  | Ak_60 | LC605373 | Ak_1 | LC605512 |
|  | 78 | Liyang, Changzhou, Jiangsu-do | China | 31.17830 | 119.52875 | Ak_61 | LC605374 |  |  |
|  |  |  |  |  |  | Ak_62 | LC605375 | Ak_1 | LC605513 |
|  |  |  |  |  |  | Ak_61 | LC605376 | Ak_1 | LC605514 |
|  | 79 | Lin'an, Hangzhou, Zhejiang-do | China | 30.31259 | 119.44897 | Ak_63 | LC605377 |  |  |
|  |  |  |  |  |  | Ak_64 | LC605378 | Ak_1 | LC605515 |
|  |  |  |  |  |  | Ak_61 | LC605379 | - |  |
| ***Anisocentropus palidus*** | | |  |  |  |  |  |  |  |
|  | 80 | Shirarutoroetoro, Shibecha, Hokkaido | Japan | 43.20233 | 144.51850 | AP_18 | LC605380 |  |  |
|  |  |  |  |  |  | AP_18 | LC605381 | Ap_2 | LC605516 |
|  |  |  |  |  |  | AP_18 | LC605382 |  |  |
|  | 81 | Asachino, Sarufutsu, Hokkaido | Japan | 45.23450 | 142.20850 | AP_10 | LC605383 | Ap_4 | LC605517 |
|  | 2 | Komasato, Chitose, Hokkaido | Japan | 42.80000 | 141.71667 | AP_10 | LC605384 |  |  |
|  |  |  |  |  |  | AP_10 | LC605385 | Ap_2 | LC605518 |
|  |  |  |  |  |  | AP_10 | LC605386 | Ap_2 | LC605519 |
|  |  |  |  |  |  | ??? | LC605387 |  |  |
|  |  |  |  |  |  | AP_10 | LC605388 |  |  |
|  |  |  |  |  |  | AP_10 | LC605389 | Ap_2 | LC605520 |
|  | 3 | Uenae, Tomakomai, Hokkaido | Japan | 42.72361 | 141.71056 | AP_10 | LC605390 | Ap_2 | LC605521 |
|  | 6 | Onumacho, Nanae, Hokkaido | Japan | 41.98333 | 140.66667 | AP_17 | LC605391 | Ap_4 | LC605522 |
|  |  |  |  |  |  | AP_17 | LC605392 |  |  |
|  | 82 | Shiritairagawa, Hanamaki, Iwate | Japan | 39.36528 | 140.97200 | Ap_15 | LC605393 |  |  |
|  |  |  |  |  |  | AP_11 | LC605394 |  |  |
|  | 83 | Hoshoguchi Yuwamemeki, Akita, Akita | Japan | 39.54836 | 140.19415 | AP_11 | LC605395 |  |  |
|  | 84 | Tsuchiyu-onsen, Fukushima, Fukushima | Japan | 37.67810 | 140.33292 | AP_22 | LC605396 | Ap_2 | LC605523 |
|  | 85 | Tamanoi, Otama, Fukushima | Japan | 37.58083 | 140.32889 | AP_10 | LC605397 | Ap_2 | LC605524 |
|  |  |  |  |  |  | AP_10 | LC605398 | Ap_2 | LC605525 |
|  | 86 | Hibara, Kitashiobara, Fukushima | Japan | 37.66889 | 140.06389 | AP_10 | LC605399 |  |  |
|  |  |  |  |  |  | AP_22 | LC605400 |  |  |
|  |  |  |  |  |  | AP_22 | LC605401 |  |  |
|  | 87 | Tadami, Tadami, Fukushima | Japan | 37.37908 | 139.26667 | Ap_16 | LC605402 |  |  |
|  |  |  |  |  |  | AP_10 | LC605403 |  |  |
|  |  |  |  |  |  | AP_14 | LC605404 |  |  |
|  | 88 | Yamamoto, Ojiya, Niigata | Japan | 37.28961 | 138.80330 | AP_19 | LC605405 |  |  |
|  |  |  |  |  |  | AP_19 | LC605406 |  |  |
|  | 89 | Irisukawa, Minakami, Gunma | Japan | 36.66857 | 138.85524 | AP_19 | LC605407 |  |  |
|  |  |  |  |  |  | AP_19 | LC605408 |  |  |
|  |  |  |  |  |  | AP_19 | LC605409 |  |  |
|  | 90 | Fujiwara, Inabe, Mie | Japan | 35.18640 | 136.46627 | Ap_8 | LC605410 |  |  |
|  |  |  |  |  |  | Ap_9 | LC605411 |  |  |
|  | 91 | Seki, Kameyama, Mie | Japan | 34.86667 | 136.40000 | Ap_7 | LC605412 | Ap_2 | LC605526 |
|  |  |  |  |  |  | Ap_4 | LC605413 | Ap_2 | LC605527 |
|  |  |  |  |  |  | Ap_8 | LC605414 |  |  |
|  | 92 | Sobura, Kaizuka, Osaka | Japan | 34.37430 | 135.42549 | AP_11 | LC605415 |  |  |
|  |  |  |  |  |  | AP_12 | LC605416 |  |  |
|  | 93 | Tamase, Takarazuka, Hyogo | Japan | 34.89550 | 135.31984 | Ap_4 | LC605417 |  |  |
|  |  |  |  |  |  | Ap_4 | LC605418 |  |  |
|  |  |  |  |  |  | Ap_4 | LC605419 | Ap_2 | LC605528 |
|  |  |  |  |  |  | Ap_6 | LC605420 |  |  |
|  |  |  |  |  |  | Ap_4 | LC605421 |  |  |
|  | 94 | Kashita, Sanda, Hyogo | Japan | 34.90770 | 135.25880 | Ap_5 | LC605422 |  |  |
|  |  |  |  |  |  | Ap_6 | LC605423 |  |  |
|  | 95 | Taga, Wake, Okayama | Japan | 34.84743 | 134.05365 | Ap_1 | LC605424 |  |  |
|  |  |  |  |  |  | Ap_1 | LC605425 |  |  |
|  |  |  |  |  |  | Ap_4 | LC605426 | Ap_1 | LC605529 |
|  | 96 | Fukuyoshi, Shoo, Okayama | Japan | 35.04599 | 134.10091 | Ap_1 | LC605427 |  |  |
|  |  |  |  |  |  | Ap_1 | LC605429 |  |  |
|  | 97 | Katsurami, Tottori, Tottori | Japan | 35.49292 | 134.17150 | Ap_1 | LC605430 |  |  |
|  |  |  |  |  |  | Ap_1 | LC605431 |  |  |
|  | 98 | Fukumaki, Nichinan, Tottori | Japan | 35.19358 | 133.24938 | Ap_1 | LC605432 |  |  |
|  |  |  |  |  |  | Ap_1 | LC605433 | Ap_2 | LC605531 |
|  |  |  |  |  |  | Ap_1 | LC605434 |  |  |
|  | 43 | Otani, Sera, Hiroshima | Japan | 34.63015 | 133.12557 | Ap_1 | LC605435 |  |  |
|  | 44 | Akitsu, Higashi-hiroshima, Hiroshima | Japan | 34.35323 | 132.81335 | Ap_1 | LC605436 |  |  |
|  |  |  |  |  |  | AP_2 | LC605437 |  |  |
|  | 99 | Yasuura, Kure, Hiroshima | Japan | 34.31882 | 132.73559 | Ap_1 | LC605438 |  |  |
|  |  |  |  |  |  | AP_13 | LC605439 |  |  |
|  |  |  |  |  |  | Ap_3 | LC605440 |  |  |
|  | 100 | Mitake, Shunan, Yamaguchi | Japan | 34.18486 | 131.90464 | AP_20 | LC605441 | Ap_3 | LC605532 |
|  |  |  |  |  |  | Ap_1 | LC605442 |  |  |
|  |  |  |  |  |  | AP_21 | LC605443 | Ap_3 | LC605533 |
|  | 55 | Innai, Usa, Oita | Japan | 33.40082 | 131.31971 | Ap_1 | LC605444 |  |  |
| ***Anisocentropus magnificus*** | | |  |  |  |  |  |  |  |
|  | 101 | Nagura, Ishigaki, Okinawa | Japan | 24.41603 | 124.16181 | Am_3 | LC605446 |  |  |
|  |  |  |  |  |  | Am_3 | LC605447 | Am_1 | LC605535 |
|  |  |  |  |  |  | Am_3 | LC605448 | Am_1 | LC605536 |
|  | 102 | Komi, Iriomote, Taketomi, Okinawa | Japan | 24.33528 | 123.91333 | Am_1 | LC605449 | Am_1 | LC605537 |
|  |  |  |  |  |  | Am_3 | LC605450 | Am_1 | LC605538 |
|  |  |  |  |  |  | Am_3 | LC605451 |  |  |
|  | 103 | Shirahama, Iriomote, Taketomi, Okinawa | Japan | 24.36861 | 123.75389 | Am_3 | LC605452 |  |  |
|  |  |  |  |  |  | Am_2 | LC605453 |  |  |

| Table S2. Primers used in this study | |  |
| --- | --- | --- |
| LCO1490 | GGTCAACAAATCATAAAGATATTGG | Folmer et al. 1994 |
| HCOoutout | GTAAATATATGRTGDGCTC | Prendini et al. 2005 |
| HexAF | ATGGCTCGTACCAAGCAGACGGC | Ogden and Whiting 2005 |
| HexAR | ATATCCTTGGGCATGATGGTGAC | Ogden and Whiting 2005 |
| The PCR protocol was: 94 °C for 1 min; 35× (94 °C for 1 min, 50 °C for 1 min, 72 °C for 1 min); 72 °C for 7 min for rTaq polymerase(TOYOBO, Osaka), or 98 °C for 1 min; 35×(94 °C for 30 sec, 50 °C for 30 sec, 72 °C for 1 min); 72 °C for 2 min for ExTaq polymerase (TAKARA, Shiga).  Reference:  Folmer, O., Black, M., Hoeh, W., Lutz, R., & Vrijenhoek, R. (1994). DNA primers for amplification of mitochondrial cytochrome c oxidase subunit I from diverse metazoan invertebrates. Molecular Marine Biology and Biotechnology, 3, 294–299.  Ogden, T. H., & Whiting, M. F. (2005). Phylogeny of Ephemeroptera (mayflies) based on molecular evidence. Molecular Phylogenetics and Evolution, 37, 625–643. https://doi.org/10.1016/j.ympev.2005.08.008  Prendini, L., Weygoldt, P., & Wheeler, W. C. (2005). Systematics of the Damon variegatus group of African whip spiders (Chelicerata: Amblypygi): Evidence from behaviour, morphology and DNA. Organisms Diversity & Evolution, 5, 203–236. https://doi.org/10.1016/j.ode.2004.12.004 | | |
|  |  |  |
|  |  |  |
|  |  |  |

| Table S3. List of specimens, sequence types, and GenBank accession numbers in Figure S2 | | | | | | | |
| --- | --- | --- | --- | --- | --- | --- | --- |
| Specimen voucher | Family | Species | EF1-α | cad | polll | IDH | COI |
| NHRS:FC1 | Calamoceratidae | *Ganonema ochraceellum* | FN600817 | FN601120 | FN600920 | FN601230 | FN601017 |
| NHRS:FC3 | Calamoceratidae | *Anisocentropus thinlin* | FN600819 | FN601122 | FN600922 | FN601232 | FN601019 |
| HRS:GE5 | Calamoceratidae | *Phylloicus lituratus* | FN600830 | FN601133 | FN600933 | FN601243 | FN601030 |
| NHRS-EN:A6 | Molannidae | *Molanna angustata* | FJ263247 | FN257671 | FN257710 | FN601139 | FJ263222 |
| NHRS:FT9 | Molannidae | *Molannodes tinctus* | FN600827 | FN601130 | FN600930 | FN601240 | FN601027 |
| NHRS:CX6 | Leptoceridae | *Condocerus paludosus* | FN600781 | FN601085 | FN600884 | FN601195 | FN600981 |
| NHRS:BH7 | Leptoceridae | *Condocerus paludosus* | FJ263266 | FN257696 | FN257735 | FN601166 | FJ263239 |
| NHRS:AE6 | Leptoceridae | *Triplectides aequalichelatus* | FN600747 | FN601045 | FN600844 | FN601153 | FN600947 |
| NHRS:AF3 | Leptoceridae | *Triplectides tigrinus* | FN600748 | FN601046 | FN600845 | FN601154 | FN600948 |
| NHRS:CU8 | Leptoceridae | *Triplectides nigripennis* | FN600767 | FN601071 | FN600870 | FN601181 | FN600967 |
| NHRS:CU9 | Leptoceridae | *Triplectides jaffueli* | FN600768 | FN601072 | FN600871 | FN601182 | FN600968 |
| NHRS:DM6 | Leptoceridae | *Trichosetodes sisyphos* | FN600789 | FN601093 | FN600892 | FN601203 | FN600989 |
| NHRS:EE8 | Leptoceridae | *Achoropsyche duodecimpunctata* | FN600800 | FN601103 | FN600903 | FN601213 | FN601000 |
| NHRS:FS4 | Leptoceridae | *Achoropsyche duodecimpunctata* | FN600821 | FN601124 | FN600924 | FN601234 | FN601021 |
| NHRS:CV1 | Leptoceridae | *Brachysetodes tripartitus* | FN600769 | FN601073 | FN600872 | FN601183 | FN600969 |
| NHRS:CV2 | Leptoceridae | *Brachysetodes bifurcatus* | FN600770 | FN601074 | FN600873 | FN601184 | FN600970 |
| NHRS:BA5 | Leptoceridae | *Ceraclea dissimilis* | FN600756 | FN601056 | FN600855 | FN601165 | FN600956 |
| NHRS:DA6 | Leptoceridae | *Ceraclea nankingensis* | FN600787 | FN601091 | FN600890 | FN601201 | FN600987 |
| NHRS:AZ6 | Leptoceridae | *Triaenodes bicolor* | FN600751 | FN601051 | FN600850 | FN601160 | FN600951 |
| NHRS:BA4 | Leptoceridae | *Triaenodes detruncatus* | FN600755 | FN601055 | FN600854 | FN601164 | FN600955 |
| NHRS:EF3 | Leptoceridae | *Triaenodes kwabena* | FN600804 | FN601107 | FN600907 | FN601217 | FN601004 |
| NHRS:FS6 | Leptoceridae | *Grumichella flaveola* | FN600822 | FN601125 | FN600925 | FN601235 | FN601022 |
| HRS:GE7 | Leptoceridae | *Grumichella flaveola* | FN600832 | FN601135 | FN600935 | FN601245 | FN601032 |
| NHRS:CW4 | Leptoceridae | *Leptocerus ousta* | FN600772 | FN601076 | FN600875 | FN601186 | FN600972 |
| NHRS:EG6 | Leptoceridae | *Leptocerus stephanei* | FN600814 | FN601117 | FN600917 | FN601227 | FN601014 |
| NHRS:GE6 | Odontoceridae | *Marilia elongata* | FN600831 | FN601134 | FN600934 | FN601244 | FN601031 |
| NHRS:FC2 | Odontoceridae | *Marilia malickyi* | FN600818 | FN601121 | FN600921 | FN601231 | FN601018 |
| NHRS CQ7 | Odontoceridae | *Barynema* sp. | FN600742 | FN601040 | FN600839 | FN601145 | FN600942 |
| EF1-α, elongation-factor 1 alpha; cad, cadherin-like protein; polll, RNA polymerase II; IDH, isocitrate dehydrogenase; COI, cytochrome oxidase subunit I; Appropriate DNA sequence data of Calamoceratidae and Molannidae, Leptoceridae, and Odontoceridae for outgroups have been used to DNA data bank of Japan (DDBJ database), and the accession numbers are given in Table SX. Sequence alignment and editing were performed for each gene separately using MEGA ver. 6.06 (Tamura et al., 2013) and CLC Workbench software (CLC bio, Aarhus). All sequence data were aligned using MAFFT v7.222 (Katoh and Standley, 2013). The alignments were determined for unique haplotypes and genotypes using the software DnaSP v4.0 (Rozas et al., 2003) prior to subsequent analysis. Phylogenetic analyses were performed by Bayesian analysis using BEAST 2 ver. 2.4.8 (Bouckaert et al., 2014). Substitution models were chosen as follows: GTR + G for the mtDNA cytochrome oxidase subunit I (350-bp) region, and nDNA elongation-factor 1 alpha (484-bp), cadherin-like protein (489-bp), RNA polymerase II (445-bp), isocitrate dehydrogenase (464-bp) regions. Bayesian MCMC simulations were run for 50 million generations, sampling every 1000 generations for the combined five regions. We selected the uncorrelated lognormal relaxed clock and a Yule tree prior. The output files were checked for convergence after removing a 10% burn-in by examining Effective Sampling Size (ESS > 200) using Tracer v1.6 (Rambaut et al., 2014), and then summarized in Tree Annotator (in BEAST package) before visualizing the resulting tree in FigTree v1.3.1 (Rambaut, 2009). | | | | | | | |
|  |  |  |  |  |  |  |  |
|  |  |  |  |  |  |  |  |
|  |  |  |  |  |  |  |  |
|  |  |  |  |  |  |  |  |
|  |  |  |  |  |  |  |  |
|  |  |  |  |  |  |  |  |
|  |  |  |  |  |  |  |  |
|  |  |  |  |  |  |  |  |
