# Supplementary material for: Phylogeography of the northernmost distributed Anisocentropus caddisflies and their comparative genetic structures based on habitat preferences
Source: Ecol Evol. 2021 Mar 30;11(9):4957–71. doi: 10.1002/ece3.7419 (PMC8093727; doi:10.1002/ece3.7419)
Supplement: Supplementary file 5 — Supplementary Material [file ECE3-11-4957-s001.docx]

**Figure S1** The estimated phylogenetic relationships (Bayes tree) of the *Anisocentropus* caddisflies based on sequenced data of the mitochondrial DNA COI region (589 bp) for estimating divergence periods of major nodes. The numbers and period values at the major nodes indicate posterior probabilities and the estimated divergence periods, respectively. The divergence period scale is shown under the phylogenetic tree. The calibration point is using the divergence period around the age that the most recent common ancestor of Molannidae and Calamoceratidae evolved between 78-132 Ma (the calibration point is shown by a star symbol; Thomas et al. 2020).

**Figure S2** The estimated phylogenetic relationships (Bayes tree) of the *Anisocentropus* caddisflies based on the combined data of 5 genetic regions (total 2,232 bp) of the mitochondrial DNA (COI region) and the nuclear DNA [EF1-α, carbamoyl-phosphate synthetase (CAD), RNA polymerase II, isocitrate dehydrogenase regions]. Lists of specimens are shown in Table S1. The estimated values of divergence ages are shown at major nodes to validate the results of divergence periods for multiple combined sequence data. The calibration point is using the divergence period~~s~~ around the age that the most recent common ancestor of Molannidae and Calamoceratidae evolved between 78-132 Ma (the calibration point is shown by a star symbol; Thomas et al. 2020).

**Figure S3** Bathymetry around Japanese Islands. The water depth is contoured and colors show 0, 200, 1000, 2000, 3000, 4000, 5000 m.
